# A Review on the Botany, Phytochemical Constituents, Pharmacological Activities, Toxicology, and Quality Control of the Medicinal Fungus *Lasiosphaera calvatia*

**DOI:** 10.3390/molecules31060948

**Published:** 2026-03-12

**Authors:** Congyao Wang, Zhuying Li

**Affiliations:** Heilongjiang University of Chinese Medicine, No. 24 Heping Street, Xiangfang District, Harbin 150040, China; wcywcy0113@163.com

**Keywords:** *Lasiosphaera calvatia*, botany, chemical constituents, pharmacology, toxicology, quality control, review

## Abstract

*Lasiosphaera calvatia* (LC), referring to the dry fruiting bodies of certain puffball fungi, has been extensively used in traditional Chinese medicine (TCM). Documented in the Mingyi Bielu, its traditional medicinal properties encompass clearing heat, detoxification, reducing swelling, and stopping bleeding. Modern applications include promoting wound healing, anti-cancer therapy, lowering blood sugar, relieving coughs, and combating HIV, among others. This comprehensive review explores the evolving scientific understanding of LC, covering its botany, traditional use, phytochemistry, pharmacology, toxicology, and quality control. A wide range of chemical components, including steroids, phenolics, volatile compounds, amino acids, polysaccharides, and polypeptides, have been isolated and identified using diverse analytical techniques. Among these, sterols (particularly ergosterol derivatives), polysaccharides, and polypeptides are considered the major bioactive constituents. The active ingredients of LC are associated with relatively few side effects, a characteristic that supports its use in pediatric populations and underscores its significant research potential. These findings validate the traditional uses of LC and lay the groundwork for further scientific exploration. The sources utilized in this study encompass Web of Science, PubMed, CNKI site, the *Chinese Pharmacopoeia*, and doctoral and master’s theses.

## 1. Introduction

*Lasiosphaera calvatia* (LC) is a general term for fungi belonging to the family Lycoperdaceae (class Basidiomycetes), which are distributed worldwide, including throughout China. Currently, 18 genera and 158 species are recognized. They commonly fruit in summer and autumn, often appearing on open grasslands or on decaying wood in moist habitats after rainfall [1]. Fungi are among the most diverse groups of organisms on this planet and play a core role in ecosystem processes and functioning [2]. As a fungal resource, LC contains numerous health-promoting compounds and is highly esteemed for its applications in the food, medicinal, and cosmetic industries [3]. Its modern applications in Traditional Chinese Medicine (TCM) are based on demonstrated antitumor, hemostatic, antibacterial, antitussive, antioxidant, and anti-inflammatory effects [4]. These wide-ranging applications are attributed to its diverse chemical profile. To date, more compounds have been identified from LC, representing a broad spectrum of chemical classes including steroids, phenolics, volatile oils, amino acids, polysaccharides, and polypeptides.

Currently, the Pharmacopeia of People’s Republic of China (2025) [5] lists two formulations containing LC: Jinsang sanjie wan and Liangjie ganmao heji. Notably, these prescriptions are used to treat hoarseness of voice, congestion and swelling of the vocal cords, chronic laryngitis, vocal cord polyps, as well as symptoms such as cold and cough. underscoring the significance of LC as an essential component in TCM. Furthermore, in clinical applications, LC is commonly combined with TCMs including *Lonicerae japonicae flos* (Jinyinhua), *Forsythiae fructus* (Lianqiao), and *Indigo naturalis* (Qingdai). For instance, Yinqiao Mabo Decoction has been proven to exert favorable therapeutic effects in the treatment of conditions such as acute tonsillitis, acute pharyngitis in children, and asthma [6]. Qingdai Mabo Decoction has been demonstrated to significantly alleviate the severity of viral pneumonia, an effect attributed to its anti-complementary, antioxidant, and anti-inflammatory activities [7,8].

The methods for species identification and component analysis of LC have evolved from traditional empirical discrimination to a modern model integrating multiple technologies. Specifically, species identification has advanced from conventional morphology-based approaches to DNA sequence-based authentication, while component analysis has developed from qualitative detection of individual components to a comprehensive qualitative and quantitative analysis system for all constituents by using hyphenated technologies such as HPLC-MS/MS. Collectively, these technological advances furnish robust scientific underpinnings for the sustainable exploitation and stringent quality control of LC as a valuable medicinal resource [9,10].

In recent years, LC has held a prominent place in pharmacies and is frequently referenced in the medical literature, with three source specials listed in the *Chinese Pharmacopoeia* (Edition 2025) [5], including *Lasiosphoera fenzlii Reich.*, *Calvatia gigantea* (*Batsch ex Pers.*) *Lloyd.* and *Calvatia lilacina* (*Mont. et Berk.*) *Lloyd*. This article focuses specifically on these three officially recognized species and conducts a systematic review to ensure the standardization, rigor, and reference value of the research content. Electronic databases including PubMed, Web of Science, and China National Knowledge Infrastructure (CNKI) were searched from their inception up to December 2025. The search was conducted using a combination of free-text words and controlled vocabulary, with key search terms including: *Lasiosphaera calvatia*, *L. fenzlii*, *C. gigantea*, *C. lilacina*, mabo, botany, taxonomy, phytochemical constituents, chemical composition, pharmacological activities, biological activities, toxicology, safety evaluation, quality control, and quality evaluation, combined by Boolean logical operators (AND/OR). Inclusion criteria were: (1) original research articles, review articles, in vitro/in vivo experimental studies, and clinical studies focusing on the botany, phytochemistry, pharmacology, toxicology, or quality control of LC; (2) articles published in Chinese or English with full text available; (3) studies with clear research purpose, standardized methods, and valid experimental or clinical data. Exclusion criteria were: (1) duplicate publications; (2) conference abstracts, letters, notes, news, and other non-peer-reviewed or incomplete documents; (3) studies irrelevant to the core theme of this review; (4) articles with serious methodological flaws, missing key data, or low research quality; (5) studies lacking clear source identification of the medicinal material LC. Two reviewers independently screened all retrieved literature by title, abstract, and full text. Any disagreements were resolved via discussion to ensure the scientificity and standardization of literature selection.

## 2. Botany and Traditional Use

**Figure 1:**
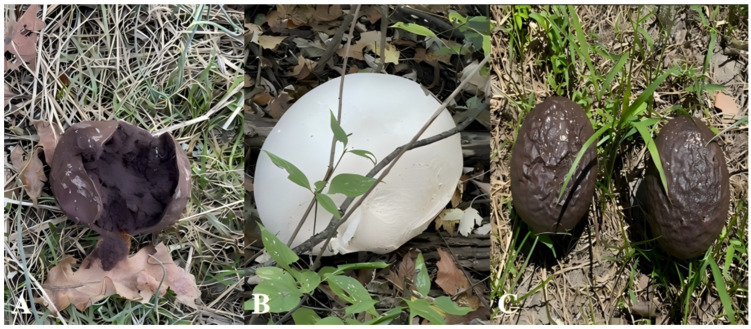
Three species of the *Lasiosphaera calvatia*: (**A**) *L. fenzlii*; (**B**) *C. gigantea*; (**C**) *C. lilacina*.

LC has a long history of medicinal use in China, owing to its broad spectrum of biological and pharmacological activities. It typically fruits after rainfall in summer and autumn and occurs singly, scattered, or in clusters. It can be found in shrublands, moist open grasslands, sandy soils, and on organic substrates such as decaying leaves, branches, and dung. Based on the search results of “Flora of China” (https://www.plantplus.cn/cn, accessed on 10 August 2025), “Chinese Plant Species Scientific Database” (DCP) (http://db.kib.ac.cn, accessed on 10 August 2025), and other websites, along with a large number of references, the distribution and botanical characteristics of the three origin LC’s have been summarized (Figure 1).

*C. gigantea* is one of the representative large fungi of the temperate grassland and meadow ecosystems in the Northern Hemisphere. It is widely distributed in the temperate grasslands and forests of Europe, northern Asia, and North America. There are sporadic records in the Southern Hemisphere, possibly due to introduction. It is widely distributed in China and shows obvious regional clustering. It is mainly concentrated in the northern, northwestern, and southwestern regions of China. *C. gigantea* produces fruiting bodies with a small or nearly absent sterile base. The remaining peridium consists of a yellowish-brown, membranous outer layer and a thicker grayish-yellow inner layer. The inner layer is smooth, hard, and brittle, and often fragments into pieces. The gleba is pale bluish-brown and feels smooth or slippery when rubbed between the fingers. Microscopically, its spores are powdery, spherical, smooth-walled or occasionally with tiny verrucae, pale bluish-yellow, with a diameter of 3.5–5 μm; the capillitia are long, concolorous with the spores, sparsely septate, and 2.5–6 μm in thickness.

*L. fenzlii* is quite common in China, and can be found almost everywhere across the country. It is more prevalent in the Yangtze River Basin and the northern regions. Its global distribution is relatively limited, mainly concentrated in East Asia, including Japan, the Korean Peninsula, the Russian Far East, and Mongolia. The fruiting body of *L. fenzlii* ranges from subglobose to nearly oblong and lacks a sterile base. Its peridium is gray-brown to yellowish-brown, papery in texture, and often breaks or sheds completely. The gleba is gray-brown to pale brown, dense yet elastic, and contains grayish-brown cotton-like filaments. When disturbed, the spores are released as a fine, dust-like powder with a soft texture. The odor is earthy, and the taste is bland. Microscopically, its capillitia are long, branched, pale brown, and 2–4.5 μm in thickness; the spores are brown, spherical, echinulate, with a diameter of 4.5–5 μm.

As an indicator species of warm, humid climates, *C. lilacina*’s distribution closely matches the range of global tropical and subtropical climate zones. It is widely distributed in tropical and subtropical regions worldwide, including Asia, Oceania (Australia and New Zealand), Africa, and parts of the Americas. In China, it is mainly distributed in the southern and southwestern regions. The fruiting body of *C. lilacina* is turbinate (top-shaped) to oblate, 5–12 cm in diameter, and has a well-developed, conspicuous sterile base. Its peridium is thin and purplish-brown, with coarse wrinkles and circular recurved depressions. The upper portion often fractures into small fragments or is partially shed, exposing the purple gleba beneath [11]. Microscopically, its spores are powdery, spherical, echinulate, with a diameter of 4–4.5 μm; the capillitia are extremely long, septate, pale in color, and 2–5 μm in thickness.

In China, the medicinal use of LC has a long history, and its origin has undergone phased changes. Before the Song Dynasty, the variety of LC was relatively single, with only *C. lilacina* available. From the Song Dynasty onward, the species of LC became increasingly diverse, including *C. gigantea*, *L. fenzlii*, etc. In TCM, LC is often combined with other Chinese medicinal herbs to make medicinal decoctions. It can also be individually ground into powder for clinical use, and its finished product, LC spore powder, is also a commonly used powder dosage form in clinical practice. Nowadays, LC remains a commonly employed TCM. Classified in TCM as having a pungent and neutral taste, it primarily affects the lung and has the effects of clearing heat, detoxifying, reducing swelling, and clearing the lungs and opening the voice, cooling the blood, and stopping bleeding [5].

Beyond its role in TCM, LC is used in Mongolian medicine and Miao medicine [12,13]. It is recorded in Mongolian medicine that its adjunct prescriptions can treat protracted menstruation and traumatic bleeding, and its pharmacognostic origin is consistent with that specified in the Pharmacopoeia. Chinese Materia Medica·Volume of Miao Medicine records that *C. gigantea* and its adjunct prescriptions are applicable for treating hematochezia, traumatic bleeding, and intestinal parasitic diseases. The single-use preparation of this herb can cure chilblains. The Secret Prescriptions of Mongolian Medicine records that applying LC powder to ulcerous lesions can achieve an immediate wound-healing effect.

## 3. Taxonomy, Nomenclature, and Classification

In the current fungal classification framework based on molecular systematics, “LC”, as defined by traditional morphology, has been verified to form not a natural monophyletic group but a typical morpho-functional group of polyphyletic origin. They are mostly placed in the class Agaricomycetes (Basidiomycota), with members widely and dispersedly distributed across multiple ordinal taxa, including Agaricales and even Boletales. Among them, the most central and common lineage, Lycoperdon (puffball genus; type species: Lycoperdon perlatum Pers. 1972) [14], is firmly classified in the family Agaricaceae. Its taxonomic status has been relatively stable since its establishment, with no notable records of synonymic changes. Because LC from different sources can be genetically distinct and thus fundamentally differ in biological properties, precise species delimitation must rely strictly on multi-locus phylogenetic analyses (e.g., ITS, LSU rRNA) combined with micromorphological characteristics, and be performed within their respective families and genera. Although the three species addressed in this paper share identical higher taxonomic ranks (Kingdom Fungi, Phylum Basidiomycota, Class Agaricomycetes, Order Agaricales, Family Agaricaceae), they belong to separate genera in this family. Such generic divergence implies potentially important differences in their biological traits.

*Calvatia gigantea* (Batsch ex Pers.) Lloyd is recorded in all editions of the Chinese Pharmacopoeia from 1977 to 2020. Its current accepted scientific name, *Calvatia gigantea* (Batsch) Lloyd, was published by American mycologist Curtis Gates Lloyd in Volume 1 of Botanical Works in 1904. It has multiple synonyms: *Lycoperdon giganteum* Batsch, *Bovista gigantea* (Batsch), *Lycoperdon bovista*, *Langermannia gigantea* (Batsch), *Calvatia maxima* (Schaeff.), *Calvatia bovista* (L.), *Lasiosphaera gigantea* (Batsch). These synonyms show considerable variation in both generic and specific epithets. The species was previously placed in four different genera: *Lycoperdon*, *Bovista*, *Langermannia/Lasiosphaera*, and *Calvatia*. The Latin epithet giganteum means “giant” and maxima means “largest”, both referring to its morphological features. It is now classified in the genus *Calvatia*.

*Calvatia lilacina* (*Mont. et Berk.*) Lloyd is also listed in the Chinese Pharmacopoeia (1977–2020). Its currently accepted name is *Calvatia lilacina* (*Mont. et Berk.*) Henn. was published by German mycologist Paul Christoph Hennings in the journal Hedwigia in 1904. One of its synonyms is Bovista lilacina Mont. & Berk., indicates the species was first described by French botanist Montagne and English naturalist Berkeley in the London Journal of Botany in 1845, before Hennings reclassified its genus. Nomenclatural changes for this species involve shifts in generic placement and author citations. The epithet *lilacina* means “lilac-colored”, named for its characteristic hue.

*Lasiosphaera fenzlii* Reich. is likewise recorded in the Chinese Pharmacopoeia (1977–2020). Its currently accepted scientific name, *Langermannia fenzlii* (Reichardt) Kreisel, was published by German mycologist Hanns Kreisel in Neue Pflanzennamen in 1962. It was first named Eriosphaera fenzlii Reichardt by Eduard Reichardt in Verhandlungen der Zoologisch Botanischen Gesellschaft in Wien in 1866, before Kreisel revised its generic assignment. The name Lasiosphaera fenzlii Reich., cited in the *2020 Chinese Pharmacopoeia*, was published by Reichardt in Plantae Novarae Austriacae in 1870. Nomenclatural changes again reflect generic reassignments, and the epithet *fenzlii* is an eponym honoring the Austrian botanist Eduard Fenzl [15].

## 4. Phytochemical Constituents

The chemical components contained in LC are quite complex, and the complexity of the LC species further exacerbates this complexity. This article merely summarizes the chemical components of *L. fenzlii*, *C. gigantea*, and *C. lilacina*. Studies have shown that the main components of LC-related medicinal herbs include steroids, phenols, volatile substances, amino acids, polysaccharides, polypeptides, and trace elements.

### 4.1. Primary Metabolites

#### 4.1.1. Fatty Acids and Their Esters

**Figure 2:**
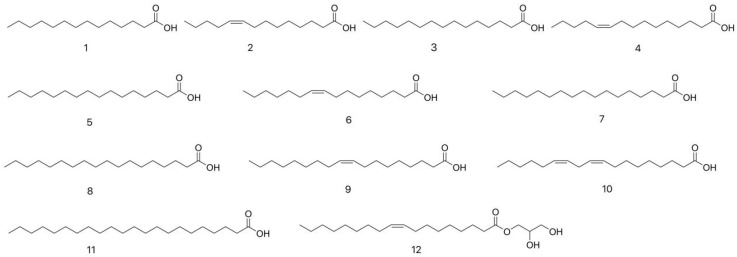
Structures of fatty acids and their ester compounds isolated from LC.

Fatty acids are a class of compounds composed of carbon, hydrogen, and oxygen. They are the main components of neutral fats, phospholipids, and glycolipids. Fatty acid esters are compounds formed by the methylation reaction of fatty acids [16]. Kivrak et al. [17] identified fatty acids from dried *C. gigantea* using gas chromatography mass spectrometry (GC–MS). Sample preparation involved grinding in liquid nitrogen, freeze–drying, methanol extraction, liquid–liquid partitioning with hexane, and derivatization. The analysis revealed the presence of 11 fatty acids: myristic acid (1), myristoleic acid (2), pentadecanoic acid (3), cis–10–pentadecenoic acid (4), palmitic acid (5), palmitoleic acid (6), heptadecanoic acid (7), stearic acid (8), oleic acid (9), linoleic acid (10), and behenic acid (11). Wang et al. [18] isolated palmitic acid (5) and stearic acid (8) from the fruiting bodies of *L. fenzlii* through petroleum ether Soxhlet extraction, silica gel column chromatography, dextran gel chromatography, and recrystallization purification. In addition, from the n-hexane extract of the dried fruiting bodies of *L. fenzlii* following ethanol extraction, Wang et al. [19] isolated and purified several compounds, based on physicochemical properties and spectral data analysis, these compounds were identified as linoleic acid (10) and benzoic acid (11). Cui et al. [20] subjected the petroleum ether extract of dried *L. fenzlii* fruiting bodies to silica gel column chromatography, leading to the isolation of compound 2,3-Dihydroxypropyloleate (12). As shown in Table 1, the fatty acid compounds’ names and molecular formulas isolated from CL are listed, and their corresponding structures are also depicted in Figure 2.

#### 4.1.2. Amino Acids

**Figure 3:**
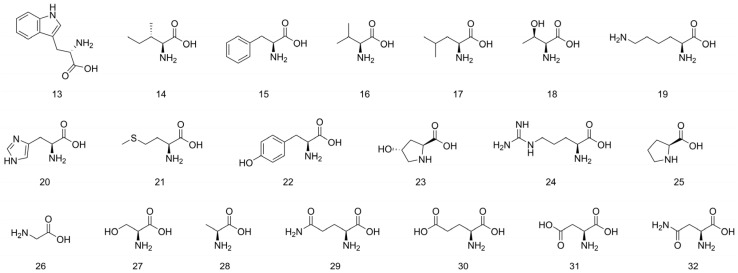
Structures of amino acid compounds isolated from LC.

Amino acids are the building blocks of protein synthesis. They are structural elements and energy sources of cells necessary for normal cell growth, differentiation, and function [21]. Kivrak et al. [22] identified 20 amino acids in dried *C. gigantea* using UPLC-MS/MS, demonstrating the presence of free amino acids in this fungus. Samples were prepared by extraction with 0.1% formic acid in water:methanol (80:20), followed by vortexing, centrifugation, and filtration prior to analysis. The detected amino acids included the essential amino acids tryptophan (13), isoleucine (14), valine (15), phenylalanine (16), leucine (17), threonine (18), lysine (19), histidine (20), and methionine (21), as well as the non-essential amino acids tyrosine (22), 4-hydroxyproline (23), arginine (24), proline (25), glycine (26), serine (27), alanine (28), glutamine (29), glutamic acid (30), asparagine (31), and aspartic acid (32). In addition, Su et al. [23] isolated phenylalanine (16) from the ethyl acetate extract of dried *L. fenzlii* fruiting bodies. The isolation was achieved by repeated column chromatography on silica gel and Sephadex LH-20, followed by HPLC purification. The identified amino acids are summarized in Table 2 and Figure 3.

#### 4.1.3. Other Primary Small Molecules

**Figure 4:**
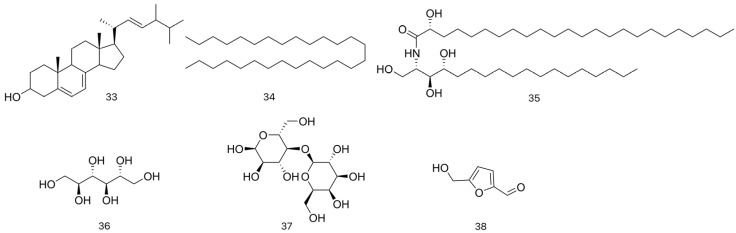
Structures of other primary small-molecule compounds isolated from LC.

As a fundamental component of fungal cell membranes, ergosterol (33) is considered a primary metabolite. It plays an essential role in maintaining membrane fluidity, integrity, and proper function, analogous to cholesterol in mammalian cells. In this study, the content in various species was quantified. Xiang et al. [24] quantified ergosterol in the dried fruiting bodies of different LC species using HPLC, revealing contents of 0.0099% in *L. fenzlii*, 0.0502% in *C. lilacina*, and 0.0123–0.1503% in *C. gigantea*, indicating significant geographical variation. Li et al. [25] further reported that ergosterol levels were consistently higher than those of its derivative ergosterone across several species (e.g., 171.58 μg/g vs. 65.29 μg/g in *C. lilacina*), consistent with its role as a primary membrane component. In addition, as shown in Table 3 and Figure 4, aromatics, aldehydes, alkenes, and other compounds were also isolated from LC in related studies. Su et al. [23] extracted 25.0 kg of dried fruiting bodies of *L. fenzlii* by refluxing with 70% ethanol three times. The combined extracts were concentrated under reduced pressure, then sequentially partitioned with petroleum ether, ethyl acetate, and *N*-butanol. The petroleum ether extract (31.0 g) was subjected to silica gel column chromatography using a gradient elution of petroleum ether-ethyl acetate, yielding six fractions. Fr.3 was purified by Sephadex LH-20 column chromatography to afford *N*-octacosane (34) (25.6 mg), which was identified by its physicochemical properties and spectral data. Cui et al. [26] crushed 5 kg of dried fruiting bodies of *L. fenzlii* and subjected the powder to Soxhlet extraction with petroleum ether. The combined extracts were concentrated under reduced pressure to yield a crude extract. This extract was then subjected to silica gel column chromatography with a gradient elution of petroleum ether-acetone. Fractions of 500 mL were collected and monitored by thin-layer chromatography (TLC). Based on TLC profiles, the fractions were pooled into six main fractions. Among them, the fourth fraction was further purified by recrystallization and was identified as (2S,3S,4R,2′R)-2-(2′-hydroxytetracosanoylamino) octadecane-1,3,4-triol (35) through physical and chemical constants as well as spectroscopic analysis. D-allitol (36) was also isolated by Cui et al. [20] from the petroleum ether extract of the dry fruiting bodies of *L. fenzlii.* Gao et al. [27] cut the dried fruiting bodies of *L. fenzlii* into pieces and refluxed them three times with 45% ethanol. The combined filtrates were concentrated and then extracted with ethyl acetate. The ethyl acetate extract was subsequently purified by silica gel column chromatography, Sephadex LH-20 gel column chromatography, and preparative HPLC to afford the compounds, which were identified as sucrose (37) and 5-hydroxymethylfurfural (38) based on their physicochemical properties and spectral data.

### 4.2. Secondary Metabolites

#### 4.2.1. Steroid Derivatives

Steroid compounds are key bioactive constituents of LC medicinal fungi, playing a critical role in their pharmaceutical potential [28]. Beyond the primary sterol ergosterol, its oxidized derivative ergosterone has attracted attention for its tissue-specific accumulation and bioactivity. Li et al. [25] found that ergosterone was predominantly distributed in mature spores and capillitia, whereas its precursor ergosterol was more abundant in mycelia. Notably, ergosterone exhibits biological activity, including the inhibition of superoxide anion radicals [29], highlighting the pharmaceutical potential of these steroid derivatives. Subsequent phytochemical investigations have led to the isolation of various bioactive steroids from LC.

Cui et al. [20,26] crushed the dried fruiting bodies of *L. fenzlii* and subjected the powder to reflux extraction with petroleum ether in a Soxhlet apparatus. The combined extracts were concentrated under reduced pressure to obtain a crude extract. This extract was then separated by silica gel column chromatography using a gradient elution of petroleum ether-acetone. The fractions were pooled based on TLC analysis, and the resulting fraction was further purified by recrystallization. The compounds were identified as ergosta-4,6,8(14),22-tetraen-3-one (39), β-sitosterol (40), ergosterol peroxide (41), ergosta-7,22(E)-dien-3β-one (42), (22E,24R)-ergosta-7,22-dien-3β-ol (43), and ergosta-7,22-diene-3,6-dione (44) through physicochemical constants and spectroscopic analysis. Following the same extraction and isolation procedure as described for *N*-octacosane (34), Su et al. [23] further isolated ergosta-7,22-diene-3β,5α,6β-triol (45) from the dried fruiting bodies of *L. fenzlii.* Wang et al. [18,30] further isolated four new steroids from the dried fruiting bodies of *L. fenzlii* through Soxhlet extraction with petroleum ether, followed by silica gel column chromatography, Sephadex gel chromatography, and recrystallization. Their structures were identified as (7,7′-biergosta-4,22-diene)-3,6,3′,6′-tetraone-8,8′-dihydroxy (48), ergosta-4,7,22-trien-3,6-dione (49), 3-hydroxyergosta-7,22-dien-6-one (50), and 5α,8α-epidoxyergosta-6,22-dien-3β-ol (51) based on spectroscopic data analysis.

Jin et al. [31] refluxed the dried fruiting bodies of *C. gigantea* with ethanol, and the combined extracts were concentrated under reduced pressure to obtain a crude extract. The extract was suspended in an appropriate amount of water, then partitioned with ethyl acetate to yield the liposoluble fraction. This fraction was subjected to silica gel column chromatography with a gradient elution of cyclohexane–ethyl acetate, yielding different crystals, which were identified as cholesteryl palmitate (46), β-sitosterol (40), ergosterol peroxide (41), and ergosta-7,22(E)-dien-3β-one (42) based on physicochemical constants and spectroscopic analysis.

**Figure 5:**
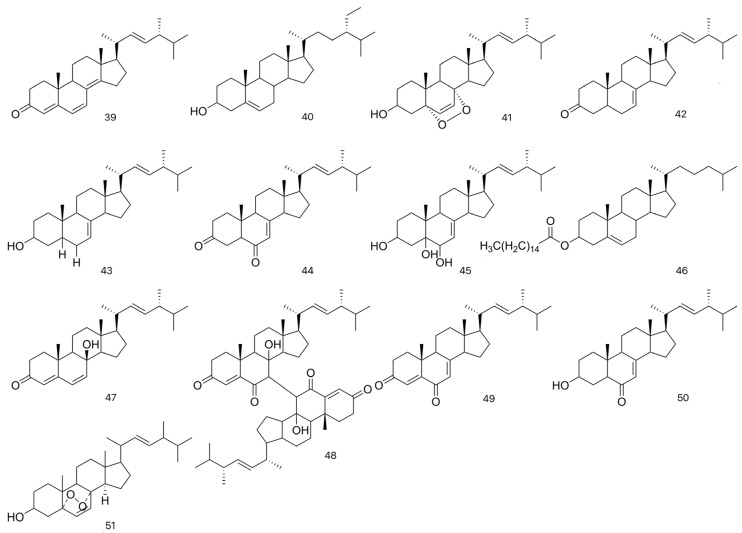
Structures of steroid derivative compounds isolated from LC.

Kawahara et al. [32] isolated two new Steroids from the dichloromethane extract of *C. lilacina*, naming them cyathiserone and cyathiserol. Structural elucidation confirmed cyathiserone as (22E,24R)-ergosta-7,22-diene-3,6-dione (44) and cyathiserol as 8β-hydroxyergosta-4,6,22-trien-3-one (47). Zeng et al. [33] extracted the dried *C. lilacina* (250 g) by heating under reflux with 95% ethanol. The extract was concentrated under reduced pressure and dried to obtain the ethanol extract, which was then partitioned sequentially with petroleum ether, ethyl acetate, and distilled water. The petroleum ether extract exhibited the greastest cytotoxicity, as determined by the MTT assay. A portion of this extract (2.4 g) was subjected to TLC, yielding seven subfractions. Among them, subfraction 7# (0.1680 g) showed the highest activity upon re-screening by MTT assay. This subfraction was analyzed by HPLC and identified as ergosta-7,22-dien-3β-one (42) by comparison with an authentic standard, marking the first confirmation of this compound in the species. These studies collectively demonstrate the structural diversity of steroids in LC medicinal fungi and lay the groundwork for further investigations into their biological activities and potential pharmaceutical applications. The identified steroid derivatives are summarized in Table 4 and Figure 5.

#### 4.2.2. Phenolics

**Figure 6:**
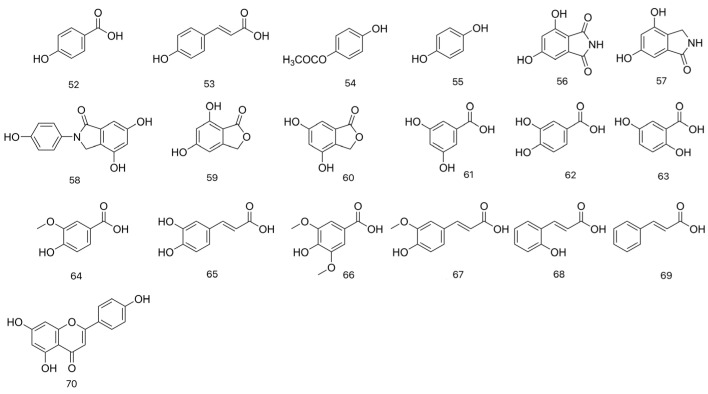
Structures of phenolic compounds isolated from LC.

Phenolic compounds, although they do not have specific nutritional value, possess special health effects. They are considered to contribute to human health owing to their hydrogen-donating properties and their ability to suppress singlet oxygen, related to their reduction power. Phenolic compounds also play an important role in food durability and the oxidative defense mechanism of biological systems in plants. [17]. Su et al. [23] extracted dried *L. fenzlii* by refluxing with 70% ethanol three times. The combined filtrate was concentrated, and the ethyl acetate extract was subjected to successive separation using silica gel column chromatography, Sephadex LH-20 gel column chromatography, and preparative HPLC, identifying compounds: p-hydroxybenzoic acid (52), p-coumaric acid (53), 4-hydroxyphenylacetate (54), p-dihydroxybenzene (55) based on their physicochemical properties and spectral data. Gao et al. [27,34] extracted the dried *L. fenzlii* with 45% ethanol under reflux, concentrated the extract, and partitioned it with ethyl acetate. The ethyl acetate fraction was subjected to repeated column chromatography (silica gel, Sephadex LH-20, ODS) and preparative HPLC, yielding compounds 4,6-dihydroxy-1H-isoindole-1, 3(2H)-dione (56), 4,6-dihydroxy-2, 3-dihydro-1H- isoindol-1-one (57), clitocybin A (58), 5,7-dihydroxy-1(3H)-isobenzofuranone (59), 4,6-dihydroxy-1(3H)-isobenzofuranone (60), 3,5-dihydroxybenzoic acid (61), which were identified by physicochemical properties and spectral data. Kivrak et al. [17] extracted dried *C. gigantea* with 80% acetone via ultrasonication. The combined extracts were evaporated, redissolved in methanol, filtered, and analyzed by UPLC-MS/MS. The analysis revealed that *C. gigantea* exhibited high phenolic content, including protocatechuic acid (62), gentisic acid (63), vanillic acid (64), caffeic acid (65), syringic acid (66), p-coumaric acid (67), ferulic acid (68), trans-2-hydroxycinnamic acid (69), and apigenin (70). As shown in Table 5 and Figure 6, the collective findings from these studies demonstrate a diverse profile of phenolic acids in LC.

#### 4.2.3. Polysaccharides, Proteins and Peptides

LC is not only rich in secondary metabolites but also contains various bioactive macromolecules such as polysaccharides and peptides. Recent studies have demonstrated that fungal polysaccharides possess significant immunostimulatory effects, while fungal peptides often exhibit unique structural features and potent bioactivity [35,36]. Herein, we describe the extraction, purification, and structural analysis of the polysaccharides and peptides obtained from LC.

Among the organic constituents of LC, polysaccharides have attracted growing research attention for their diverse bioactivities, with *C. gigantea* polysaccharide (CGP) serving as a representative example [37]. CGP can be fractionated into three components, namely CGP I-1, CGP II, and CGP III, all of which are composed of mannose, glucose, and galactose with distinct molar ratios (1.22:11.28:3.92, 1.74:26.45:0.36, and 1.06:9.90:3.29, respectively). Chemically, CGP II and CGP III are classified as acidic polysaccharides with uronic acid contents of 17.2% and 20.5%, respectively, while CGP I-1 is a neutral polysaccharide. Structurally, all three CGP fractions are predominantly pyranose-form polysaccharides with 1→4 glycosidic linkages as the main backbone and a small proportion of 1→6 linkages; CGP I-1 exists as a mixture of α- and β-anomers, whereas CGP II and CGP III are dominated by β-anomers [38]. Zhao et al. [39] investigated the factors influencing the extraction yield of polysaccharides from the dried fruiting body of *C. gigantea* using an orthogonal experimental design. The results showed that the order of influencing factors from greatest to least was: extraction temperature > solid–liquid ratio > extraction time > number of extraction cycles. Based on the range analysis, the optimal extraction conditions were determined as follows: 0.5 h extraction time, 3 extraction cycles, 100 °C extraction temperature, and a solid–liquid ratio of 1:15. A validation experiment conducted under these conditions yielded an extraction rate of 1.792%, indicating a relatively high yield.

Furthermore, as major water-soluble components of LC, proteins and peptides have also been isolated and characterized. Meng et al. [40] cut dried *L. fenzlii* fruiting bodies and extracted them with ethanol-water under reflux. The filtrate was concentrated, applied to a macroporous resin column, and eluted with ethanol. The eluate was concentrated to obtain a white crude peptide extract that tested positive for cyclic peptides and was identified as WLIP by physicochemical and spectral analysis. They then quantified WLIP contents in samples from different origins or commercial cities using HPLC. The results demonstrated that the WLIP content ranged from 0.0142 to 0.0594 mg/g. In addition, the initial isolation of calvacin, a mucoprotein from the dried fruiting body of *C. gigantea*, was reported by Kim et al. [41]. Subsequent studies confirmed its antitumor activity and employed purification methods involving ammonium sulfate precipitation and chromatography [42]. The polysaccharides, proteins and peptides isolated from LC and their corresponding sources are summarized in Table 6.

### 4.3. Inorganic Elements

Inorganic elements play a significant role in the quality evaluation of TCM. They are not only involved in plant root nutrition and physiological metabolism but also serve as constituent factors in secondary metabolite biosynthesis. Variations in the types and contents of inorganic elements can directly affect the efficacy of medicinal herbs, making them important indicators of TCM quality [43]. In LC, Mn, Fe, Mg, Ca, and K have been identified as characteristic inorganic elements that play key roles in its growth and development. Studies have shown that these elements exhibit certain correlations during plant absorption and accumulation. In different LC species, eight pairs of elements exhibit synergistic effects in absorption, and most elements show cooperative interactions that may contribute to enhanced efficacy. Furthermore, inorganic elements can combine with organic compounds in TCM to form coordination complexes, thereby influencing therapeutic effects [44].

Yue et al. [45] analysed the inorganic elemental compositions of fenzlii, C. gigantea, and C. lilacina using ICP-MS and identified manganese, iron, magnesium, calcium, and potassium as the characteristic inorganic elements of LC. The results indicated that the inorganic element composition was generally similar across species, but their contents varied considerably. Among the major elements, the average mass fractions of Mg, P, K, and Ca were substantially higher than those of other elements, with K exhibiting the highest average content (19,361.873 mg/kg), followed by P (8269.081 mg/kg), Ca (2356.177 mg/kg), and Mg (1641.577 mg/kg). Interspecific comparisons revealed that *L. fenzlii* had the highest average K and Ca contents, *C. gigantea* had the highest Mg content, and *C. lilacina* had the highest P content.

Regarding trace elements, Fe showed the highest average mass fraction (3169.630 mg/kg), with considerable variation among samples, reaching 7622.425 mg/kg in sample DM-1. This was followed by Zn (284.447 mg/kg), Mn (94.510 mg/kg), and Cr (14.309 mg/kg), while Se displayed the lowest average content (6.717 mg/kg). In terms of species characteristics, *L. fenzlii* showed the highest average Zn content, *C. gigantea* exhibited the highest Mn and Fe contents, and *C. lilacina* demonstrated the highest Cr and Se contents.

For heavy metal elements, the average mass fractions of Cd, Hg, Pb, As, and Cu were 1.501, 2.986, 6.091, 14.058, and 101.071 mg/kg, respectively. Among these, *L. fenzlii* showed the highest average As content, while *C. lilacina* exhibited the highest average contents of Cd, Hg, Pb, and Cu.

PCA results demonstrated that samples of the same species clustered together, indicating relatively small differences in inorganic element contents within species. However, distinct variations were observed among different species: *L. fenzlii* was enriched in K, Ca, and Zn; *C. gigantea* showed enrichment in Mg, Mn, and Fe; and *C. lilacina* was characterized by higher contents of P, Cr, and Se. It is speculated that these interspecific differences in inorganic element contents may be associated with various ecological factors in their respective growing environments.

### 4.4. Volatile Components: Composition and Influencing Factors

The LC contains a large number of volatile components, including alkenoids, hydrocarbons, ketones, aldehydes, alcohols, etc. Its essential oil has been reported to have good antibacterial activity [46].

The composition and content of LC volatile components are regulated by multiple factors, with fruiting body maturity being a key variable. Kivrak et al. [17] analyzed fresh *C. gigantea* samples mixed with anhydrous magnesium sulfate after incubation at 90 °C for 30 min using headspace gas chromatography–mass spectrometry (HS-GC/MSD), and identified 7 aldehydes, 4 alcohols, 1 ketone, 1 acid, 1 alkene, and 1 furan by mass spectral library matching. You et al. [47] extracted volatile oils from the fruiting bodies of *C. gigantea* at different maturation stages using steam distillation and identified the components by GC-MS. A total of 14 and 16 compounds were detected in the volatile oils of immature and mature fruiting bodies, respectively. Specifically, the essential oil from mature *C. gigantea* was dominated by saturated fatty acids and alkanes, while the immature specimen also contained sesquiterpenoids such as cedrol, caryophyllene, and caryophyllene oxide, indicating that fruiting body maturity significantly influences the composition and content of volatile components.

In addition to fruiting body maturity, extraction methods play a crucial role in determining the profile of LC volatile components. Xu et al. [48] were the first to extract the essential oil of *L. fenzlii* by steam distillation and analyze it by GC-MS. A total of 89 compounds were separated, of which 49 were identified, accounting for 70.713% of the total volatile oil. The major components were acenaphthylene (16.836%), ar-curcumene (7.800%), caryophyllene oxide (5.448%), beta-tumerone (2.814%), and beta-cadinene (2.771%). Xiang et al. [49] further compared the volatile components of LC extracted by traditional steam distillation with those obtained via lipid-soluble solvent ultrasonic extraction. The main components of the essential oil from steam distillation were 2,2′-methylenebis-(4-methyl-6-tert-butylphenol) and 7-amino-1,4-dimethylpyrimido [4,5-c]pyridazine-3,5-(1H,2H)-dione; hexadecanoic acid ethyl ester was the main component in LC samples extracted by ethyl acetate ultrasonic extraction, while 2,2′-methylenebis-(4-methyl-6-tert-butylphenol) was the primary component in the essential oil extracted by ether ultrasonic extraction.

Furthermore, interspecific differences in volatile components between LC species have been confirmed by comparative analyses. Li et al. [50] analyzed the volatile components of the fruiting bodies of *C. gigantea* and *C. lilacina* using headspace solid-phase microextraction combined with infrared spectroscopy and GC-MS. The results showed that the volatile components of *C. gigantea* included 40 alkanes, 6 alkenes, 5 aromatic hydrocarbons, 2 alcohols, 1 lipid, 1 ketone, and 5 ethers; in contrast, those of *C. lilacina* consisted of 57 alkanes, 3 alkenes, 3 aromatic hydrocarbons, 3 alcohols, 4 lipids, 1 ketone, 2 ethers, 1 aldehyde, and 2 heterocyclic compounds. Both species were rich in alkanes, but *C. lilacina* exhibited greater diversity in volatile component classes.

**Figure 7:**
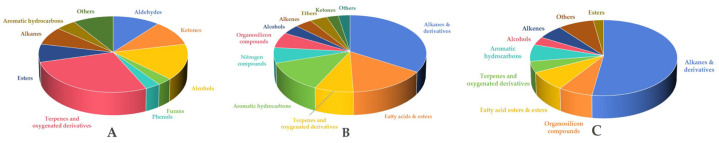
The contents of various LC volatile components ((**A**). *L. fenzlii*; (**B**). *C. gigantea*; (**C**). *C. lilacina*).

Collectively, the compounds contained in different types of LC bulbs are different (Figure 7). *L. fenzlii* is rich in terpenoids and characteristic C8 compounds, which form the basis of its potent antibacterial and anti-inflammatory activities. These components act via multiple pathways—such as disrupting microbial membranes and inhibiting inflammatory mediator release–providing direct lead compounds for treating respiratory/skin-soft tissue infections and adjuvant antitumor therapy, and serving as optimal raw materials for novel antibacterial/anti-inflammatory drugs. The essential oil of *C. gigantea* shows significant developmental dependence, making its medicinal value dynamic and regulable. Immature stages are rich in sesquiterpenoids, while mature stages are dominated by alkanes and fatty acid esters (likely involved in physical barrier repair and metabolism regulation). This requires strict standardization of harvest time for clinical use and enables differentiated medication strategies for different disease stages (e.g., acute infection, chronic repair). In contrast, *C. lilacina*’s essential oil is mainly simple-structure long-chain alkanes, with relatively weak direct pharmacological activity. Its medical value lies not in the therapeutic effects of the volatile components themselves, but in the physical barrier properties of these alkanes—supporting its potential use in developing novel pharmaceutical excipients (e.g., sustained-release carriers, ointment bases) and wound dressings.

In conclusion, the aforementioned chemical composition studies demonstrate that although *L. fenzlii*, *C. gigantea*, and *C. lilacina* are taxonomically distinct species, their medicinal materials are systematically composed of chemically diverse components with extensive bioactivities (Table 7). These components encompass three categories: primary metabolites essential for sustaining life (e.g., amino acids, polysaccharides, and inorganic elements); characteristic secondary metabolites responsible for exerting core pharmacological effects (e.g., steroids and phenols); and volatile components that possess unique ecological and organoleptic properties. Notably, the major bioactivities identified in the table—including anti-inflammatory, antioxidant, immunomodulatory, antitumor, and antibacterial effects—are not mediated by a single component independently. Instead, they arise from a multi-component and multi-target synergistic network formed by different classes of compounds.

## 5. Toxicology

Toxicological studies on *C. gigantea* have revealed a favorable safety profile alongside moderate, selective cytotoxicity. The crude extract of *C. gigantea* demonstrated low toxicity in the Artemia salina lethality assay, with a median lethal concentration (LC_50_) > 1000 μg/mL, which is classified as non-cytotoxic according to the Meyer criteria [9]. This finding was corroborated by an MTT assay on normal Vero cells, in which the median cytotoxic concentration (CC_50_) was 337.4 μg/mL, substantially higher than that of the positive control cyclophosphamide (CC_50_ = 8.71 μg/mL) [51]. These data provide experimental support for its historical use as an edible fungus, as documented in various ethnobotanical records [41,51]. In terms of cytotoxicity quantification, the XTT (2,3-bis(2-methoxy-4-nitro-5-sulfophenyl)-2H-tetrazolium-5-carboxamide) assay showed that when cells were treated with *C. gigantea* extract at concentrations ranging from 25 μg/mL to 2 mg/mL in a time- and dose-dependent manner, the half-maximal inhibitory concentration (IC_50_) was 500 μg/mL at 72 h of treatment [52], though it should be noted that the mature spores of *C. gigantea* may act as airborne allergens and trigger type I hypersensitivity reactions, a key point requiring attention in safety assessment [53].

In summary, *C. gigantea*, *L. fenzlii*, and *C. lilacina* showed favorable safety profiles at conventional doses. Most toxicity-related observations were associated with hypersensitivity rather than inherent acute or systemic toxicity, supporting the reasonable safety of these fungal species as medicinal materials.

## 6. Quality Control

As an important medicinal fungus, the accurate identification and quality control of LC have long been focal points and challenges in the field. Traditional reliance on morphological expertise is prone to subjectivity and environmental variability, necessitating the development of a more robust, multi-technology integrated system that combines morphological, microscopic, molecular, and physicochemical approaches [54,55].

Modern systematic identification emphasizes the convergence of genotype and phenotype information. At the molecular level, DNA barcoding based on the internal transcribed spacer (ITS) sequence has become a standard tool for species discrimination. Studies have successfully distinguished genuine LC (including *L. fenzlii*, *C. gigantea*, *C. lilacina*) from adulterants by analyzing genetic distances and phylogenetic trees, where authentic species form distinct monophyletic clades [56,57]. Although ITS2 secondary structure data provide auxiliary information, their phylogenetic resolution is generally lower than that of primary sequence data; thus, they are best used in conjunction with other markers [58]. Furthermore, multi-gene analyses (e.g., ITS + LSU) are advancing the revision of macro-classification systems within Lycoperdaceae, refining phylogenetic relationships and generic boundaries [1].

Physicochemical identification, which directly reflects the chemical basis of medicinal materials, remains widely applied in standards for fungal medicines due to its operational simplicity. For LC, common methods include ignition tests, color reactions, and particularly thin-layer chromatography (TLC) with reference materials. Recently, chemical fingerprinting techniques (e.g., HPLC-MS) have gained prominence for their high information capacity and specificity. For instance, protein fingerprints of *L. fenzlii* have been established, effectively classifying samples into quality grades and accurately grouping authentic geoherbal samples [34].

Quantitative assessment based on characteristic active components is central to quality evaluation. For LC, steroids such as ergosterol and ergosterone serve as key markers. HPLC analyses reveal that ergosterol is typically more abundant and concentrated in hyphae and cortex, whereas ergosterone predominates in spores and capillitia [25]. This distribution suggests that the content and ratio of these compounds can inform species attribution and quality assessment. Optimized extraction and chromatographic methods (e.g., employing ethyl acetate and acetonitrile-formic acid mobile phases) have been established for the accurate quantification of ergosterol across different species and origins [24].

In conclusion, quality control for LC has evolved into a synergistic model integrating genotypic authentication (e.g., DNA barcoding), phenotypic and chemical profiling (e.g., TLC, fingerprinting), and quantitative analysis of characteristic constituents (e.g., ergosterol). This multi-dimensional framework ensures more reliable identification and quality evaluation. Future advancements will likely incorporate more sophisticated spectroscopic and spectrometric technologies, driving the development of more efficient, accurate, and comprehensive standards for the standardized application of LC medicinal fungi.

## 7. Pharmacological Effects

LC, as a TCM in our country, has a pungent and neutral nature and taste, and has the effects of clearing the lungs, relieving sore throat, and stopping bleeding. Modern pharmacological studies have shown that LC has anti-tumor, hemostatic, antibacterial, expectorant, antioxidant, and anti-inflammatory effects. These effects are closely related to the complex chemical composition of these substances (such as steroids, polysaccharides, and peptides). This chapter will systematically review the research progress on the pharmacological effects of LC, including its mechanism of action, active ingredients, and relevant experimental evidence, to provide a reference for its clinical application and subsequent research.

### 7.1. Antitumor Effects

**Figure 8:**
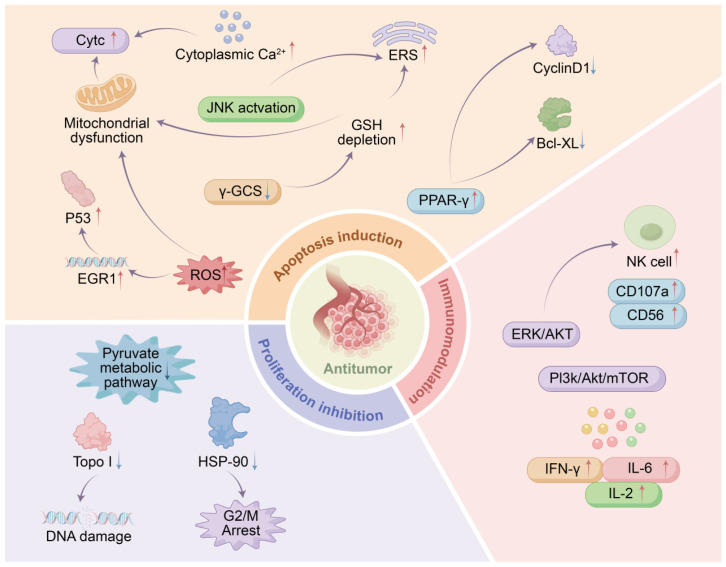
Mechanisms of LC in Antitumor Activity. ↑ means increase; ↓ means decrease.

In recent years, the antitumor effects of LC have garnered significant research interest. As summarized in Figure 8, LC exerts its antitumor activity through multiple mechanisms, including inducing tumor cell apoptosis, inhibiting proliferation, and modulating the immune system.

#### 7.1.1. Induction of Tumor Cell Apoptosis

Apoptosis is a form of programmed cell death that maintains cellular homeostasis and primarily involves the mitochondrial-mediated intrinsic pathway and the death receptor–mediated extrinsic pathway. The intrinsic apoptotic pathway is often activated by stimuli such as DNA damage and oxidative stress [59]. Meng et al. [36] found that WLIP, isolated from *L. fenzlii*, exhibited significant antiproliferative effects on K562 cells. WLIP promoted apoptosis, induced G0/G1-phase arrest, and downregulated the expression of Bcl-xL and cyclin D1. To verify whether PPAR-γ is a direct target of WLIP, the researchers further employed a luciferase reporter gene assay to detect PPAR-γ activity, using rosiglitazone as a positive control. The results demonstrated that 10 μmol/L WLIP significantly activated PPAR-γ, suggesting that its antitumor effect may be, at least in part, mediated through modulation of the PPAR-γ signaling pathway.

Studies have shown that the purified water-soluble protein extract of *C. lilacina* can reduce the viability of SW480 cells and THP-1 cells [60]. Tsay et al. [61] further demonstrated that this protein extract specifically induces apoptosis in human colorectal cancer SW480 cells while sparing normal cells. The underlying mechanism involves inhibition of γ-glutamylcysteine synthetase (γ-GCS)–mediated glutathione (GSH) biosynthesis, leading to intracellular GSH depletion and triggering cell death. Although this process is accompanied by increased production of reactive oxygen species (ROS) and upregulation of Bax protein, experimental evidence indicates that both are secondary events. This study established that GSH depletion is the principal mechanism underlying the anticancer effect of the protein extract of *C. lilacina*, providing a new strategy for the treatment of colorectal cancer.

Eroğlu et al. [52] treated human lung cancer A549 cells with 500 μg/mL MeOH crude extract of *C. gigantea* fruiting bodies for 72 h and found that the extract induced cell-cycle arrest and apoptosis by downregulating the expression of CCND1, CCND2, CDK4, Akt, and Bcl-2, while upregulating Bax, p53, caspase-3, and caspase-9. Regarding extract fractions, Zeng et al. [33] further partitioned the 95% ethanol extract of *C. lilacina* into petroleum ether, ethyl acetate, and aqueous fractions and found that the petroleum ether fraction exerted the strongest inhibitory effect on human breast cancer MDA-MB-231 cells. The mechanism was associated with enhanced ROS generation, which induced mitochondrial dysfunction, promoted cyt c release into the cytoplasm, and triggered apoptosis through Apaf-1-dependent activation of the caspase cascade.

#### 7.1.2. Inhibition of Tumor Cell Proliferation

Uncontrolled proliferation is a key biological characteristic of tumor cells, and inhibiting this ability is a central strategy in cancer therapy [62]. Cytotoxic agents can specifically target rapidly dividing tumor cells, thereby exerting selective killing effects on cancer cells. Multiple studies have shown that extracts from LC exhibit significant cytotoxicity against a variety of tumor cell lines—including breast cancer, cervical cancer, liver cancer, prostate cancer, colorectal cancer, ovarian cancer, endometrial cancer, and neuroblastoma—effectively inhibiting tumor cell proliferation and growth in a time- and concentration-dependent manner.

Cell cycle regulation represents an important approach to suppressing tumor proliferation [63]. Calvacin, a basic mucoprotein extracted from the spores of LC via water extraction, has been confirmed to possess antitumor activity. Hou et al. [42] found that calvacin downregulates topoisomerase I (TopoI) activity, thereby impairing DNA synthesis and causing damage to human gastric cancer SGC-7901 cells. Additionally, this component suppresses the expression of heat shock protein 90 (HSP-90), thereby impairing its chaperone function in binding to cell cycle-related protein kinases. This leads to the suppression of cyclin-dependent kinase activity, resulting in cell cycle arrest at the G2/M phase. Such arrest further compromises the DNA damage repair process during the G2/M phase, exacerbating cellular damage and ultimately leading to tumor cell death. The half-maximal inhibitory concentration (IC50) of calvacin in SGC-7901 cells was measured to be 29.74 µg/mL by the MTT method. The IC50 value measured by the SRB method was 27.77 µg/mL, and the trend of proliferation inhibition was consistent. This further proves that it has a significant inhibitory effect on gastric cancer cells.

Ergosterol, a phytosterol commonly found in mushrooms, acts as a precursor to vitamin D_2_. Nilkhet et al. [64] found that ergosterol significantly reduced the viability of breast cancer cells, induced G0/G1 phase arrest, and inhibited spheroid formation. Further investigations demonstrated that ergosterol exerted its function by targeting the Wnt/β-catenin signaling pathway, specifically through suppressing the AKT/GSK-3β axis to promote the phosphorylation and proteasomal degradation of β-catenin, reduce its nuclear translocation, and thereby downregulate downstream target genes such as c-Myc and cyclin D1. In addition, ergosterol downregulated critical metabolic pathways (e.g., steroid hormone biosynthesis) in MDA-MB-231 cells. This study is the first to clarify the impact of ergosterol on the β-catenin pathway and confirms that, in addition to its anti-proliferative activity, ergosterol can also inhibit cancer stemness, providing new insights into its anti-tumor potential.

Ergosterol peroxide (EP) is a key active substance; in vitro experiments showed that EP inhibited various cancer cells (e.g., HeLa cells) with an IC_50_ range of 13.6–17.2 μg/mL, while showing very low toxicity to normal cells even at 50 μg/mL, indicating significant selective killing effect associated with the peroxide group in its chemical structure (derivatives lacking this group lose antitumor activity) [65]. More importantly, EP has a prominent synergistic effect with chemotherapy drug paclitaxel (PTX): a low-dose combination (0.05 μg/mL PTX + 12.5 μg/mL EP) can inhibit 80% of HeLa cell growth, whereas a single dose of PTX needs to be 10 times higher (0.5 μg/mL) to achieve the same effect, and mechanistic studies revealed EP significantly increases PTX accumulation in cancer cells, amplifying PTX’s cytotoxic effect on cancer cells without affecting normal cells. As the core active component of *L. fenzlii*, EP takes the peroxide group as the key pharmacological moiety, not only selectively disrupting cancer cell homeostasis but also acting as a chemosensitizer to enhance chemotherapy efficacy by increasing intracellular chemotherapeutic drug concentration and reducing systemic toxicity of the combined regimen, providing new insights for developing low-toxicity and highly effective combined chemotherapy strategies [33].

Regarding polysaccharide components, Zhao et al. [39] reported that *C. gigantea* alcohol-soluble and alcohol-extractable polysaccharides both exhibit strong inhibitory effects on the two selected tumor cells. However, the inhibitory effect of alcohol-soluble polysaccharides on Siha cells is higher than that of alcohol-extractable polysaccharides, with an inhibition rate as high as 52.6%, while that of alcohol-extractable polysaccharides is only 25.84%. On the contrary, for MDA cells, the inhibitory effect of alcohol-extractable polysaccharides is significantly higher than that of alcohol-soluble polysaccharides. The highest inhibition rate of alcohol-extractable polysaccharides is 84.05%, while that of alcohol-soluble polysaccharides is only 65.95%. Therefore, the inhibitory effect of mabu polysaccharides on different tumor cell lines is selective. Wu et al. [66] further showed that the acidic polysaccharide CGP-II from *C. gigantea*, at a dose of 200 mg/(kg·d), exhibited an inhibition rate of 48.4% against S180 sarcomas. This polysaccharide also demonstrated DPPH free radical scavenging capacity and oxygen radical absorbance capacity, suggesting that its antioxidant activity may contribute to its antitumor mechanism. Furthermore, steroidal constituents from LC have shown potential in suppressing proliferation. Wang et al. [18] found that ergosta-4,7,22-triene-3,6-dione at 50 μg/mL, inhibited the proliferation of chronic myeloid leukemia K562 cells by 64%. Cui et al. [26] also reported that ergosta-7,22-diene-3β-one could inhibit the proliferation of human hepatoma Bel-7402 cells and glioma C6 cells.

#### 7.1.3. Immunomodulation

Immunotherapy is a therapeutic strategy that harnesses the body’s natural immune system to combat cancer [67,68]. Interferon-gamma (IFN-γ), the only member of the type II interferon family, is a water-soluble dimeric cytokine produced exclusively by activated T lymphocytes and NK cells. It exhibits multiple biological functions, including antiviral, immunomodulatory, and antitumor activities [69]. Zhang et al. [70] demonstrated that polysaccharides derived from LC significantly increased the spleen index, thymus index, NK cell proliferation rate, and splenic lymphocyte proliferation stimulation index in tumor-bearing mice, indicating enhanced innate immune function. Furthermore, these polysaccharides elevated serum levels of cytokines such as IFN-γ, IL-6, and IL-2, effectively activating macrophages and promoting the growth and differentiation of lymphocytes. These mechanisms highlight the critical immunomodulatory role of LC polysaccharides in antitumor immunity. Additionally, inhibition of the PI3K/Akt/mTOR signaling pathway has been identified as another important mechanism through which LC polysaccharides suppress breast cancer growth.

### 7.2. Wound Healing Promotion

**Figure 9:**
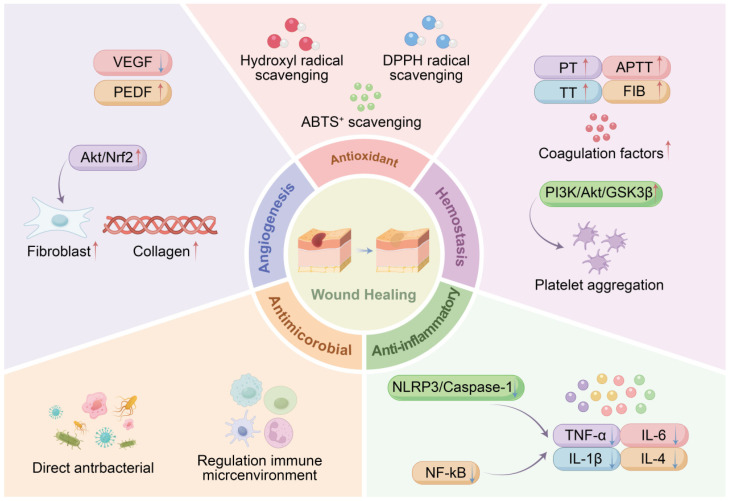
Mechanisms of LC in Promoting Wound Healing. ↑ means increase; ↓ means decrease.

LC has a long history of use in promoting wound healing. Studies have shown that puffball facilitates wound repair across the hemostatic, inflammatory, proliferative, and remodeling phases of wound healing, by exerting effects such as hemostasis, anti-inflammation, bacteriostasis, antioxidation, blood glucose reduction, and promotion of fibroblast proliferation and collagen secretion. (show in Figure 9).

#### 7.2.1. Hemostasis

Hemorrhage represents a major challenge in trauma care, particularly under extreme conditions such as battlefields, where excessive bleeding accounts for up to 50% of fatalities [71]. Bleeding time and coagulation time are key indicators for evaluating the procoagulant and hemostatic effects of drugs and are closely associated with platelet function and capillary integrity. The coagulation process is primarily regulated through both the intrinsic and extrinsic pathways [72].

In traditional medicine, one of the most common uses of LC is as a wound dressing, with its sporogenous tissue demonstrating effective hemostatic properties. Yang et al. [73] reported that both the sporocarp and peridium of *L. fenzlii*, as well as their ethyl acetate and *N*-butanol extracts, exhibited significant hemostatic activity, with the peridium showing a greater blood-adsorption capacity than the sporocarp. Further research by Li et al. [74] demonstrated that the crude extract of *C. gigantea* and *C. lilacina*, prepared from dried fruiting bodies via prolonged hot-water extraction (95 °C) followed by concentration, exhibits hemostatic activity through a multi-pathway synergistic mechanism. It not only significantly affects coagulation-related indicators, including PT, APTT, TT, and FIB, but also increases levels of endogenous coagulation factor XII and exogenous coagulation factors X and VII, thereby activating both the intrinsic and extrinsic coagulation pathways. Meanwhile, it downregulates t-PA levels and upregulates PAI-1 levels to inhibit fibrinolysis, and its high-dose group can elevate platelet counts. In contrast, the aqueous extract of *C. lilacina* exerts hemostatic effects mainly by activating the intrinsic coagulation pathway: it remarkably shortens APTT and TT, increases the levels of endogenous coagulation factors XII and VIII, and also significantly raises t-PA content, with its high-dose group also capable of increasing platelet counts. Further studies have revealed that both aqueous extracts of *C. gigantea* and *C. lilacina* can significantly enhance the phosphorylation of PI3K, Akt, and GSK3β, and thus participate in the hemostatic process by promoting platelet aggregation through the PI3K/Akt/GSK3β signaling pathway [50].

In a clinical study, Yang et al. [75] evaluated the efficacy of an LC ointment in the treatment of mixed hemorrhoids and confirmed its definite therapeutic effects in alleviating symptoms such as hematochezia, hemorrhoid prolapse, perianal itching, and constipation.

In summary, LC has demonstrated remarkable hemostatic efficacy in clinical practice and is commonly used as a powder, mycelial sponge, or ointment for the treatment of traumatic bleeding, epistaxis, and gastrointestinal hemorrhage. However, pharmacological studies on its individual active components remain limited and warrant further in-depth investigation.

#### 7.2.2. Anti-Inflammatory Effects

The inflammatory response is a critical phase of wound healing, during which neutrophils and macrophages release large amounts of pro-inflammatory mediators in the early stages. When inflammation is excessive or persistent, impairing the clearance of inflammatory cells and cytokines, the wound-healing process can be significantly delayed. Therefore, anti-inflammatory intervention plays an important role in wound management.

Yang et al. [76] found that an LC ointment inhibited the NLRP3/Caspase-1 inflammatory signaling pathway and significantly reduced the expression of key inflammatory cytokines, including Interleukin-1 beta (IL-1β) and Tumor Necrosis Factor-alpha (TNF-α) in rabbit wounds infected with *Escherichia coli*, thereby promoting wound healing. Xiang et al. [77] further demonstrated that the water extract of *C. gigantea* exhibited notable anti-inflammatory activity in acute inflammation models—including xylene-induced ear edema and egg white-induced paw edema—as well as in a cotton pellet-induced granuloma model of subacute inflammation.

LC preparations have also shown therapeutic potential in the treatment of intestinal inflammation. Yang et al. reported that an LC enema solution significantly reduced the levels of pro-inflammatory cytokines such as TNF-α and IL-6 in a mouse model of ulcerative colitis, effectively suppressing the inflammatory response and alleviating colonic tissue damage [78]. Zhang et al. [79] established an ulcerative colitis model using oxazolone combined with trinitrobenzene sulfonic acid and found that treatment with an LC suppository markedly improved the colonic mucosal pathology. The protective mechanism was closely associated with reduced levels of inflammatory cytokines—including TNF-α and IL-4—in colonic tissues and serum, as well as inhibition of the NF-κB signaling pathway.

Li et al. [80] investigated the phytochemical profile and diabetic wound-healing activity of CLP2, an ethanol extract fraction isolated from *C. lilacina*, and found that CLP2 contains 14 bioactive components, with ergosterol being the most abundant at 837.3 μg/mg. In vivo experiments demonstrated that CLP2 significantly accelerates wound healing in db/db diabetic mice by promoting re-epithelialization, inhibiting M1 macrophage polarization, downregulating the transcriptional levels of pro-inflammatory cytokines, and enhancing the expression of anti-inflammatory factors. In vitro assays further revealed that CLP2 can stimulate the proliferation and migration of mouse skin fibroblasts, suppress the secretion of pro-inflammatory cytokines associated with M1 macrophages, and facilitate M2 macrophage polarization. Collectively, both in vivo and in vitro experiments confirmed that CLP2 exerts a pro-healing effect on diabetic wounds by inhibiting M1 macrophage polarization and promoting M2 macrophage polarization, thereby accelerating the transition of wounds from the inflammatory to the proliferative phase. Thus, CLP2 has great potential as a therapeutic agent for diabetic wound management.

Additionally, He et al. [81] applied single-cell RNA sequencing to deeply explore how *C. lilacina* spores reduced the number of monocytes to decrease the inflammatory state of the granulomatous tissue of patients with anal fistula, while increasing the communication between IL6 + macrophages and fibroblasts, thus accelerating the transition from the inflammatory phase to the proliferative phase of the wound.

Collectively, these studies indicate that LC effectively inhibits the release of pro-inflammatory mediators by regulating different inflammatory signaling pathways and exhibits significant anti-inflammatory effects in diverse models. Its multiple formulations demonstrated therapeutic potential for both skin wounds and intestinal inflammation, highlighting LC as a candidate multi-target anti-inflammatory agent.

#### 7.2.3. Antimicrobial Effects

Bacterial biofilm formation is a major factor contributing to the persistence of chronic wounds. Bacterial exotoxins can trigger sustained inflammatory responses, promoting excessive release of proteases and reactive oxygen species by inflammatory cells, which further damages regenerating tissues. Common pathogenic bacteria in chronic refractory wounds include *Staphylococcus*, *Corynebacterium*, *Pseudomonas aeruginosa*, *Escherichia coli*, and *Streptococcus* spp. [82].

Zhang et al. [83] systematically evaluated the antimicrobial activity of various polar extracts of *Calvatia gigantea.* Their results showed that ethanol, ethyl acetate, diethyl ether, mycelial, and fermentation broth extracts all exhibited varying degrees of inhibitory effects against Staphylococcus aureus, Pseudomonas aeruginosa, and *Escherichia coli*. You et al. [84] compared the antimicrobial efficacy of multiple extracts from *C. gigantea.* at different maturation stages using the disk-diffusion method. All tested components inhibited the growth of *E. coli* ATCC 8099 and S. aureus ATCC 6538. Among them is the chloroform extract of immature *C. gigantea.* showed the strongest inhibitory effect at a concentration of 50 mg/mL, with inhibition rates of 57.57% and 50.12%, respectively, and a minimum inhibitory concentration (MIC) of 12.5 mg/mL for both strains. The acetone extract of mature *C. gigantea.* also demonstrated considerable activity at the same concentration, with inhibition rates of 50.59% and 45.47%, and an MIC of 12.5 mg/mL. Furthermore, Khan et al. [85] found that *C. gigantea* methanolic extract has strong anti-MDR pathogen activity. The ZOI against *K. pneumoniae* was 23 mm, and the minimum was 11 mm against *E. cloacae*.

Notably, the ecological balance of the wound microbiota plays a crucial regulatory role in the healing process. Studies suggest that commensal bacteria can promote tissue repair through mechanisms such as immunomodulation, making them a focus of interest in the treatment of chronic wounds [86]. Ding et al. [87] found that intervention with *C. gigantea.* significantly increased the diversity of the wound microbiota, reduced the abundance of pathogenic bacteria, such as S. aureus, and simultaneously enriched commensals, such as *E. coli*, resulting in a more balanced microbial community structure. This microbiota-modulating effect further promoted macrophage polarization toward the M2 anti-inflammatory phenotype, enhanced the proliferation and migration of keratinocytes and fibroblasts, and thereby improved epithelialization and tissue barrier regeneration.

This dual mechanism of direct antibacterial action combined with regulation of the immune microenvironment endows LC with unique advantages in the treatment of chronic wounds.

#### 7.2.4. Antioxidant Effects

The wound-healing process is closely associated with the level of reactive oxygen species (ROS). While appropriate ROS levels contribute to bactericidal activity, signal transduction, and regulation of inflammatory responses, excessive ROS can lead to protein denaturation, loss of cellular function, and exacerbation of inflammation, thereby impeding tissue repair [88].

Kivrak et al. [17] systematically evaluated the antioxidant capacity of various solvent extracts (methanol, hexane, ethyl acetate, and aqueous) from *C. gigantea* using cupric-reducing antioxidant capacity, DPPH free-radical-scavenging, and ABTS^+^ decolorization assays. The results indicated that all extracts exhibited cupric-ion-reducing antioxidant activity comparable to that of the positive controls, BHA and α-tocopherol. The ethyl acetate extract showed a clear dose-dependent DPPH radical–scavenging effect, reaching 86% at 800 μg/mL, which was superior to both BHA and α-tocopherol at the same concentration. At 200 μg/mL, it achieved an ABTS^+^ scavenging rate of 89%, whereas α-tocopherol at the same concentration showed a scavenging rate of 86%.

Further research by Li et al. [89] revealed that the crude polysaccharide from the liquid-fermented mycelium of *Calvatia gigantea* exhibited a positive correlation between its scavenging capacity against DPPH radicals, hydroxyl radicals, and superoxide anions and its concentration within the experimental range. At 100 μg/mL, the scavenging rates for DPPH, OH, and O_2_^−^ were 32%, 56%, and 48%, respectively. Zhu et al. [90] also reported that the hydroxyl-radical-scavenging capacity of *C. lilacina* polysaccharides gradually increased with concentration within the range of 0.0884–0.4420 mg/mL.

Taken together, these results indicate that the antioxidant activity of puffball primarily stems from the pronounced free-radical-scavenging ability of its various extracts and polysaccharide components. By directly neutralizing reactive oxygen species such as DPPH radicals, ABTS^+^, and hydroxyl radicals, these constituents effectively mitigate oxidative damage to wound cells and thereby create a more favorable microenvironment for tissue repair.

#### 7.2.5. Promotion of Angiogenesis

Fibroblasts serve as the primary repair cells during the proliferative phase of wound healing, playing crucial roles in collagen secretion, extracellular matrix formation, and granulation tissue formation. Studies have shown that functional abnormalities in fibroblasts are a key factor contributing to delayed healing in chronic refractory wounds, particularly in diabetic skin ulcers [91]. Active components derived from LC have been found to effectively promote fibroblast proliferation and collagen synthesis, offering new insights for accelerating wound repair.

Yang et al. [92] demonstrated that an ointment prepared from the sporocarp of *L. fenzlii*, when applied to chronic skin ulcers in rabbits, promoted angiogenesis in the wound by down-regulating vascular endothelial growth factor (VEGF) expression and significantly up-regulating pigment epithelium-derived factor (PEDF) expression. He et al. [93] further elucidated the mechanism of LC spore powder in a diabetic rat ulcer model, showing that it activates the Akt/Nrf2 signaling pathway, enhances the expression of antioxidant genes, and significantly promotes angiogenesis and collagen formation.

Meng et al. [94] isolated and purified a novel homogeneous water-soluble polysaccharide, CGPA1, from the fruiting bodies of *C. gigantea*, which is primarily composed of galactose, methylgalactose, mannose, xylose, and glucose. Both in vitro and in vivo experiments confirmed that CGPA1 promotes fibroblast migration, facilitates skin re-epithelialization, increases collagen deposition, and significantly accelerates the healing of acute wounds. Additionally, Shi et al. [95,96] found that the ethanol extract of LC exerted a significant proliferative effect on fibroblasts cultured in vitro, with collagen production increasing in a dose-dependent manner, reaching up to 247% of the blank control level at the highest tested concentration.

In summary, LC promotes wound healing through multiple mechanisms, including hemostasis (regulating the coagulation pathway), anti-inflammation (inhibiting inflammatory pathways such as NLRP3/Caspase-1), antibacterial (directly inhibiting bacteria and regulating the wound microbiota), antioxidation (clearing free radicals), and angiogenesis (activating Akt/Nrf2 pathways, etc.). Different extracts and active components of LC play key roles at each stage of wound repair, making it a multi-target candidate for chronic wound treatment.

### 7.3. Hepatoprotective Effect

LC has shown protective potential against both chemical and alcohol-induced liver injury. Its active components can mitigate hepatic damage through multiple mechanisms, including regulation of oxidative stress, suppression of inflammatory responses, and modulation of the gut microbiota, thereby preserving the structural and functional integrity of liver tissue. Zhang et al. [97] reported that ergosterone significantly reduced serum AST and γ-GT, as well as hepatic ALT and malondialdehyde (MDA) levels, in mice with alcohol-induced liver injury, while increasing the activities of prealbumin (PA) and superoxide dismutase (SOD) in liver tissue. Ergosterone also reversed alcohol-induced alterations in gut microbiota composition at both the phylum and genus levels, suggesting that its hepatoprotective effect may be closely related to the regulation of the gut–liver axis. Mu et al. [98] further confirmed that an ethanol extract of *C. lilacina* mycelia reduced AST, ALT, and total bilirubin (TB) levels in mice with alcoholic liver injury, enhanced SOD activity, and inhibited MDA generation, thereby attenuating sustained lipid peroxidation in hepatocytes. In addition, the extract significantly decreased hepatic levels of inflammatory cytokines, including IL-18, IL-1β, and TNF-α, thereby alleviating liver inflammation. Li et al. [89] demonstrated that a crude polysaccharide from submerged mycelia (CPSM) of *C. gigantea* exerted reparative effects on CCl_4_-induced liver injury by significantly lowering AST and ALT levels and improving liver, spleen, and thymus indices, indicating both immunomodulatory and antioxidant properties. Su et al. [99] found that β-stiosterol can improve liver function in mice with liver injury induced by realgar, alleviate liver inflammatory responses and oxidative stress, and protect liver tissues. Its mechanism of action may involve inhibition of PI3K/Akt signaling pathway activation. In summary, the multi-component, multi-target actions of LC provide an experimental basis for its further development as an adjunctive therapy for liver diseases.

### 7.4. Other Pharmacological Effects

In addition to the aforementioned activities, LC has demonstrated other pharmacological properties, including antitussive, hypoglycemic, and anti-HIV effects. With respect to glucose and lipid metabolism, Ogbole et al. [9] found that the methanolic extract of *Calvatia gigantea* significantly reduced blood glucose levels by 29.3% in alloxan-induced diabetic rats. Li et al. [25] further investigated the protective mechanism of ergosterol against diabetic kidney injury and showed that it significantly decreased triglyceride (TG), total cholesterol (TC), serum uric acid (SUA), and serum creatinine (Scr) levels in streptozotocin-induced diabetic mice. This renoprotective effect was attributed to inhibition of PI3K, Akt, NF-κB p65, and IκBα phosphorylation, thereby alleviating renal pathological damage. Regarding effects on the respiratory system, Zuo et al. [100] reported that *L. fenzlii* significantly inhibited xylene-induced ear edema in mice and prolonged cough latency in guinea pigs, suggesting combined anti-inflammatory and antitussive activities. In antiviral research, Ma et al. [101] evaluated LC in an HTLV-1–persistently infected MT-4 cell model challenged with HIV-1 at a TCID50 dose. Their results showed that both aqueous and methanolic extracts of *L. fenzlii* exhibited anti–HIV-1 activity, with IC50 values of 250 μg/mL and 125 μg/mL, and CC0 values of 500 μg/mL and 250 μg/mL, respectively, indicating therapeutic potential within a reasonable safety window.

In summary, different species of LC have unique chemical components and corresponding advantageous pharmacological research directions. Table 8 lists the mechanism of pharmacological action. Among them, *C. gigantea* has been the most extensively studied, with research covering aspects such as anti-tumor, wound-healing, liver protection, and hypoglycemic effects. Steroid compounds (especially ergosterol) are one of the most crucial pharmacologically active substances shared by Ganoderma fungi, and are associated with various core activities such as anti-tumor, liver protection, and regulation of blood sugar. Polysaccharides are another major class of common active components, reported in *C. gigantea* and *C. lilacina*, mainly associated with immune regulation, anti-tumor, and antioxidant effects. In addition, each species has unique, highly active components, such as WLIP isolated from *L. fenzlii* and Calvacin protein isolated from *C. gigantea*, which exhibit high activity.

## 8. Conclusions and Future Perspectives

As a traditional medicinal fungus with a long history of use, LC has been recognized for its remarkable therapeutic value from ancient times to the present day. This review systematically summarizes the research progress on three major medicinal species of LC, namely *L. fenzlii*, *C. gigantea*, and *C. lilacina*, covering their botanical characteristics, traditional applications, chemical components, pharmacological activities, toxicology, and quality control. Research findings indicate that LC is rich in a variety of bioactive components, including steroids (e.g., ergosterol), polysaccharides, polypeptides, phenols, and amino acids. These components collectively form the material basis for its multi-target and multi-functional pharmacological effects. Modern pharmacological studies have confirmed that LC and its bioactive components exhibit significant anti-tumor, wound-healing promotion, hepatoprotection, cough relief, hypoglycemia and antivirus activities. These findings not only provide scientific evidence for its traditional efficacy but also reveal its broad potential for application in modern medicine.

However, current research still faces several urgent problems and challenges, and future studies should focus on the following directions.

First, the systematization of chemical components and quality standards should be considered. Given LC’s complex chemistry and variations in content across species, regions, and batches, refined norms for cultivation, harvesting, and processing are needed. A multidimensional evaluation system—integrating morphology, DNA barcoding (e.g., ITS sequences), chemical fingerprinting (e.g., HPLC-MS profiles), and key marker quantification (e.g., ergosterol)—should be developed to ensure uniformity of medicinal materials.

Second, bioactive component mechanism exploration should be performed. Most studies rely on crude extracts, lacking analysis of individual compounds’ structure-activity relationships, targets, and signaling networks. Future work should isolate high-activity monomers (e.g., steroid derivatives, defined polysaccharides) and use molecular/network pharmacology to clarify mechanisms, especially in tumor microenvironment regulation and tissue repair.

Third, pharmacokinetics and formulation development should be performed. In vivo absorption, distribution, metabolism, and excretion data of bioactive components are scarce, limiting dosage optimization. Systematic pharmacokinetic studies are urgently required to clarify bioavailability, enabling new delivery systems (e.g., nano-systems, topical dressings) to boost targeting and efficacy.

Fourth, safety evaluation and clinical translation should be considered. Though preliminary data suggest safety, long-term/genetic toxicity and drug interaction evaluations are needed. Rigorous clinical trials (backed by preclinical evidence) should verify efficacy in diabetic foot ulcers, adjuvant tumor therapy, etc., advancing it to evidence-based clinical use.

Fifth, sustainable resource use is essential. Artificial cultivation and submerged fermentation should be developed to secure raw materials, while synthetic biology could produce scarce high-value components (e.g., specific peptides), enabling deeper utilization.

In summary, LC is a treasure trove connecting traditional wisdom and modern science. Through interdisciplinary integration and technological innovation, in-depth exploration of its chemical and pharmacological properties will surely accelerate the modernization and internationalization of LC, lay a solid foundation for the development of innovative drugs and functional products with independent intellectual property rights, and enable it to play a greater role in advancing human health.

## Figures and Tables

**Table 1:** Fatty acid and their ester compound names and molecular formulas isolated from LC.

Compound No.	Chemical Constituents	Molecular Formula	Source	Parts	Refs.
1	myristic acid	C_14_H_28_O_2_	B	a	[17]
2	myristoleic acid	C_14_H_26_O_2_	B	a	[17]
3	pentadecanoic acid	C_15_H_30_O_2_	B	a	[17]
4	cis-10-pentadecenoic acid	C_15_H_28_O_2_	B	a	[17]
5	palmitic acid	C_16_H_32_O_2_	A, B	a	[17,18]
6	palmitoleic acid	C_16_H_30_O_2_	B	a	[17]
7	heptadecanoic acid	C_17_H_32_O_2_	B	a	[17]
8	stearic acid	C_18_H_36_O_2_	A, B	a	[17,18]
9	oleic acid	C_18_H_34_O_2_	B	a	[17]
10	linoleic acid	C_18_H_32_O_2_	A, B	a	[17,19]
11	behenic acid	C_22_H_44_O_2_	A, B	a	[17,19]
12	2,3-Dihydroxypropyl oleate	C_21_H_40_O4	A	a	[20] ^1^

^1^ Footer: A. *L. fenzlii*; B. *C. gigantea*; a. Fruiting body.

**Table 2:** Amino acid compound names and molecular formulas isolated from LC.

Compound No.	Chemical Constituents	Molecular Formula	Source	Parts	Refs.
13	tryptophan	C_11_H_12_N_2_O_2_	B	a	[22]
14	isoleucine	C_6_H_13_NO_2_	B	a	[22]
15	valine	C_5_H_11_NO_2_	B	a	[22]
16	phenylalanine	C_9_H_11_NO_2_	A, B	a	[22,23]
17	leucine	C_6_H_13_NO_2_	B	a	[22]
18	threonine	C_4_H_9_NO_3_	B	a	[22]
19	lysine	C_6_H_14_N_2_O_2_	B	a	[22]
20	histidine	C_6_H_9_N_3_O_2_	B	a	[22]
21	methionine	C_5_H_11_NO_2_S	B	a	[22]
22	tyrosine	C_9_H_11_NO_3_	B	a	[22]
23	4-hydroxyproline	C_5_H_9_NO_3_	B	a	[22]
24	arginine	C_6_H_14_N_4_O_2_	B	a	[22]
25	proline	C_5_H_9_NO_2_	B	a	[22]
26	glycine	C_2_H_5_NO_2_	B	a	[22]
27	serine	C_3_H_7_NO_3_	B	a	[22]
28	alanine	C_3_H_7_NO_2_	B	a	[22]
29	glutamine	C_5_H_10_N_2_O_3_	B	a	[22]
30	glutamic Acid	C_5_H_9_NO_4_	B	a	[22]
31	aspartic Acid	C_4_H_7_NO_4_	B	a	[22]
32	asparagine	C_4_H_8_N_2_O_3_	B	a	[22] ^1^

^1^ Footer: A. *L. fenzlii*; B. *C. gigantea*; a. Fruiting body.

**Table 3:** Other compound names and molecular formulas isolated from LC.

Compound No.	Chemical Constituents	Molecular Formula	Source	Parts	Ref.
33	ergosterol	C_28_H_44_O	A, B, C	a, b	[24]
34	*N*-octacosane	C_28_H_58_	A	-	[23]
35	(2S,3S,4R,2′R)-2-(2′-hydroxytetracosanoylamino) octadecane-1,3,4-triol	C_42_H_85_NO_6_	A	a, b	[26]
36	D-allitol	C_6_H_14_O_6_	A	a, b	[26]
37	sucrose	C_12_H_22_O_11_	A	a	[27]
38	5-hydroxymethylfurfural	C_6_H_6_O_3_	A	a	[27] ^1^

^1^ Footer: A. *L. fenzlii*; B. *C. gigantea*; C. *C. lilacina*; a. Fruiting body; b. Mycelial fermentation broth.

**Table 4:** Steroid compound names and molecular formulas isolated from LC.

Compound No.	Chemical Constituents	Molecular Formula	Source	Parts	Refs.
39	ergosta-4,6,8(14),22-tetraen-3-one	C_28_H_40_O	A, C	a	[26,29]
40	β-stiosterol	C_29_H_50_O	A, B	a	[26,31]
41	ergosterol peroxide	C_28_H_44_O_3_	A, B	a	[20,26]
42	ergosta-7,22(E)-dien-3β-one	C_28_H_44_O	A, B, C	a, b	[20,26,32]
42	(22E,24R)-ergosta-7,22-dien-3β-ol	C_28_H_46_O	A	a	[20,26]
44	ergosta-7,22-diene-3,6-dione	C_28_H_42_O_2_	A, C	a	[20,26,32]
45	ergosta-7,22-diene-3β,5α,6β-triol	C_28_H_46_O_3_	A	-	[23]
46	cholesteryl palmitate	C_43_H_76_O_2_	B	a	[31]
47	8β-hydroxyergosta-4,6,22-diene-3-one	C_28_H_42_O_2_	A, C	-	[20,32]
48	(7,7′-biergosta-4,22-diene)-3,6,3′,6′-tetraone-8,8′-dihydroxy	C_56_H_82_O_6_	A	-	[18]
49	ergosta-4,7,22-trien-3,6-dione	C_28_H_40_O_2_	A	-	[18]
50	3-hydroxyergosta-7,22-dien-6-one	C_28_H_44_O_2_	A	-	[18]
51	5α,8α-epidoxyergosta-6,22-dien-3β-ol	C_28_H_44_O_3_	A	a	[18,23,30] ^1^

^1^ Footer: A. *L. fenzlii*; B. *C. gigantea*; C. *C. lilacina*; a. Fruiting body; b. Mycelial fermentation broth.

**Table 5:** Phenolic compound names and molecular formulas isolated from LC.

Compound No.	Chemical Constituents	Molecular Formula	Source	Parts	Refs.
52	p-hydroxybenzoic acid	C_7_H_6_O_3_	A, B	a	[17,23]
53	p-coumaric acid	C_9_H_8_O_3_	A, B	a	[17,23]
54	4-hydroxyphenyl acetate	C_8_H_8_O_3_	A	-	[23]
55	p-dihydroxybenzene	C_6_H_6_O_2_	A	a	[17,23]
56	4,6-dihydroxy-1H-isoindole-1,3(2H)-dione	C_8_H_5_NO_4_	A	a	[27,34]
57	4,6-dihydroxy-2,3-dihydro-1H-isoindol-1-one	C_8_H_7_NO_3_	A	a	[27,34]
58	clitocybin A	C_14_H_11_NO_4_	A	a	[27,34]
59	5,7-dihydroxy-1(3H)-isobenzofuranone	C_8_H_6_O_4_	A	a	[27,34]
60	4,6-dihydroxy-1(3H)-isobenzofuranone	C_8_H_6_O_4_	A	a	[27,34]
61	3,5-dihydroxybenzoic acid	C_7_H_6_O_4_	A	a	[27,34]
62	protocatechuic acid	C_7_H_6_O_4_	B	a	[17]
63	gentisic acid	C_7_H_6_O_4_	B	a	[17]
64	vanillic acid	C_8_H_8_O_4_	B	a	[17]
65	caffeic acid	C_9_H_8_O_4_	B	a	[17]
66	syringic acid	C_9_H_10_O_5_	B	a	[17]
67	ferulic acid	C_10_H_10_O_4_	B	a	[17]
68	p-coumaric acid	C_9_H_8_O_3_	B	a	[17]
69	trans-2-hydroxycinnamic acid	C_9_H_8_O_3_	B	a	[17]
70	apigenin	C_15_H_10_O_5_	B	a	[17] ^1^

^1^ Footer: A. *L. fenzlii*; B. *C. gigantea*; a. Fruiting body.

**Table 6:** Polysaccharide, protein and peptide names and sources isolated from LC.

Compound No.	Chemical Constituents	Source	Parts	Ref.
71	CGP	B	-	[38]
72	WLIP	A	-	[40]
73	calvacin	B	-	[41] ^1^

^1^ Footer: A. *L. fenzlii*; B. *C. gigantea*.

**Table 7:** Correlation between Chemical Categories, Their Distribution in Species, and Their Main Pharmacological Activities.

Category	Representative Compounds	Source	Main Bioactivities	Refs.
Steroids	Ergosterol, Ergosterone, β-Sitosterol, etc.	A, B, C	anti-inflammatory, antioxidant, and immune-regulating	[20,23,24,26,27,34,37]
Phenolics	p-hydroxybenzoic acid, coumaric acid, protocatechuic acid, etc.	A, B	antioxidant, antibacterial, anti-inflammatory	[17,23,27,34]
Fatty Acids and Esters	Palmitic acid, stearic acid, linoleic acid, 2,3-dihydroxypropyl oleate, etc.	A, B	regulating blood lipids, anti-inflammation	[17,18,19,20]
Amino Acids	Essential amino acids (tryptophan, isoleucine, etc.), non-essential amino acids (tyrosine, glycine, etc.)	A, B	nutritional supplementation, immune regulation	[22,23]
Polysaccharides and Polypeptides	CGP, WLIP, Calvacin	A, B, C	immune enhancement, anti-tumor, anti-bacterial	[36,37,41]
Inorganic Elements	K, Ca, Zn, Mg, Mn, Fe, Se, etc.	A, B, C	maintain body metabolism, synergistically enhance activity	[16]
Volatile Components	Alkanes, alkenes, alcohols, ketones, aldehydes, aromatic hydrocarbons, etc.	A, B, C	antibacterial and bacteriostatic	[46,47,48,49] ^1^

^1^ Foot: A. *L. fenzlii*; B. *C. gigantea*; C. *C. lilacina*.

**Table 8:** Mechanisms of pharmacological action of LC.

Pharmacology	Mechanism	Species	Extracts/Compounds	Chemical Profile/Compound Class	Diseases	Model		Pathway	Effects	Ref.
						In Vitro	In Vivo			
Antitumor	apoptosis-inducing	*C. lilacina*	*C. lilacina* extract	Petroleum ether extract of fruiting bodies.	breast cancer	MDA-MB-231	/	/	↑ Bax, c-PARP, p-p53 ↓Bcl-2, caspase-3,	[33]
		*L. fenzlii*	WLIP	White powder; cyclopeptide, molecular mass 1125 Da	chronic myelogenous leukemia	K562	/	PPAR-γ	↓ Bcl-xL, CCND1 ↑ PPAR-γ	[36]
		*C. gigantea*	*C. gigantea* extract	MeOH crude extract of fruiting bodies	lung cancer	A549	/	Akt	↓ CCND1, CCND2, CDK4, Akt, Bcl-2 ↑ Bax, p53, caspase-3, caspase-9	[52]
		*C. lilacina*	*C. lilacina* extract	Partially purified water-soluble protein extract (>10 kDa); major protein bands 11–70 kDa (SDS-PAGE)	colon cancer	SW480	/	/	↓ γ-GCS ↑ Bax, ROS	[60]
		*C. lilacina*	*C. lilacina* protein	Crude water-soluble protein extract, protein fractions > 10 kDa	colon cancer	SW480, HT-29, DLD-1	/	ERS, JNK	↑ P-eIF2α, caspase-4, caspase-9, ATF3, ATF4, CHOP, GRP78, JNK	[61]
	Inhibition of Tumor Cell Proliferation	*L. fenzlii*	ergosta-4,7,22-triene-3,6-dione	Colorless acicular crystals; molecular weight 408.3028 Da.	chronic myeloid leukemia	K562	/	/	↑ tumor suppression	[18]
		*C. gigantea* *C. Lilacina* *L. fenzlii*	2,3-dihydroxypropyloleate	White solid; molecular weight 356 Da.	Liver cancer, glioma	Bel-7402 C6	/	/	↑ tumor suppression	[26]
		*C. gigantea*	*C. gigantea* extract	Crude polysaccharide extract from LC fruiting bodies; maximum extraction yield 1.792%.	cervical cancer	Siha cells	/	/	↑ tumor suppression	[39]
		*C. gigantea*	calvacin	Mucoprotein; molecular weight ~ 17 kDa.	gastric cancer	SGC-7901	/	/	↓ HSP-90, TopoI	[42]
		*C. gigantea* *C. Lilacina* *L. fenzlii*	ergosterol	Most abundant characteristic sterol in LC; molecular weight 396.3392 Da.	breast cancer	MCF-7, MDA-MB-231	/	Wnt/β-catenin AKT/mTOR	↓ c-Myc, CCND1, p-Akt, p-4EBP1, USP47, USP9x	[64]
		*C. gigantea*	CGP-II	Acidic polysaccharide extract; uronic acid content 17.2%.	sarcomas	/	S180	/	↑ tumor suppression	[66]
	Immunomodulation	*C. gigantea*	CGP	Light yellow powder crude polysaccharide; yield 1.58%, total polysaccharide content 80.6%.	breast cancer	/	BALB/c	PI3K/Akt/mTOR	↓ IFN-γ, IL-6, IL-2, p-PI3K, p-Akt, p-mTOR	[70]
Wound Healing	Hemostasis	*C. gigantea* *C. Lilacina*	*C. gigantea*/*C. Lilacina* extracts	Complex crude extract; dried fruiting bodies, hot water extraction (95 °C) and concentration.	bleeding	/	Kunming mouse	PI3K/Akt/GSK3β	↓ t-PA ↑ PLT, p-GSK3β/GSK3β	[50]
		*C. gigantea* *C. Lilacina*	*C. gigantea*/*C. Lilacina* extracts	Complex crude extract; dried fruiting bodies, hot water extraction (95 °C) and concentration.	bleeding	/	Kunming mouse	/	↓ PT, APTT, TT ↑ FIB	[74]
	Anti-inflammatory	*C. gigantea*	*C. gigantea* extract	Aqueous decoction crude extract (1 g crude drug/mL).	ears swollen, paw swollen	/	SD mouse	/	↓ swollen, writhe	[75]
		*C. lilacina*	*C. lilacina* extract	Crude ethanol extract.	diabetic wounds	mouse skin fibroblasts, RAW246.7	db/db mice	/	↓ IL-6, IL-1β, CD86, Nos2, TNF- α ↑ Arg1, Mrc1, IL-10, TGF- β	[80]
	Antimicrobial	*C. gigantea*	*C. gigantea* extract	Crude extract; dried fruiting bodies, ultrasound-assisted water extraction.	ears swollen	*G. Staphylococcus*, *E. coli* Brucella, *P. aeruginosa*	Kunming mouse	/	↓ ears swollen MIC = 15 mg·mL^−1^, anti-inflammatory, analgesic	[83]
		*C. gigantea*	*C. gigantea* extracts	Petroleum ether, chloroform, acetone, methanol extracts, and volatile oil.	bacteria	*E. coli*, *S. aureus*	/		↓ ATCC8099, ATCC6538	[84]
		*C. gigantea*	*C. gigantea* extract	Crude extract; sporophore powder, 95% ethanol sonication, concentration and lyophilization.	diabetic ulcer wounds	/	db/db mice	/	↓ IL-1β, IL-18, IL-6, TNF-α, CD86, Nos2, IL-1α ↑ Pcna, Mcam, VEGFA, Collα1, Col3α1	[87]
	Antioxidant	*C. gigantea*	*C. gigantea* extracts	Ethyl acetate extract; total phenolics 41.36 µg PE/mg; total flavonoids: 8.55 µg QE/mg.	/	/	/	/	↑ DPPH, ABTS+	[17]
		*C. gigantea*	*C. gigantea* extracts	methanol, ethyl acetate, *N*-hexane, aqueous and pure water extracts.	bacterial infection	/	/	/	↓ *Acinetobacter baumannii*, *Staphylococcus aureus*, *Pseudomonas aeruginosa*, *Klebsiella pneumonia*, *Proteus mirabilis*, *Enterobacter cloacae* and *Escherichia coli*.	[85]
		*C. lilacina*	*C. Lilacina* water-soluble polysaccharide	Water-soluble crude polysaccharide extract; hot water maceration and concentration.	/	/	/	/	↑ hydroxyl-radical-scavenging capacity	[90]
	Angiogenesis	*C. gigantea*	CGPA1	Homogeneous polysaccharide fraction; total sugar content 91.22%, weight-average molecular weight ~ 11.69 kDa.	wound	MSFs	Balb/c mice	/	proliferative effect on fibroblasts cultured in vitro, with collagen production increasing in a dose-dependent manner	[94]
Hepatoprotective		*C. gigantea*	CPSM	Crude polysaccharide extract from mycelia.	acute alcoholic liver injury	/	Kunming mouse	/	↓ AST, ALT ↑ scavenging abilities of DPPH free radicals, hydroxyl radicals and superoxide anions	[89]
		*C. lilacina* *C. gigantea*	ergosterone	Fungal-specific steroidal ketone; molecular weight 396.339 Da.	acute alcoholic liver injury	/	C57BL/6 mice	/	↓ AST, γ-GT, ALT, MDA ↑ PA, SOD	[97]
		*C. lilacina*	*C. lilacina* extracts	Water and ethanol extracts of mycelia.	acute alcoholic liver injury	/	C57BL/6 mice	/	↓ ALT, AST, MDA, IL-18, IL-1β, TNF-α, TB ↑ SOD	[98]
		*C. gigantea* *L. fenzlii*	β-stiosterol	A stigmastane-type phytosterol with a C-24 ethyl side chain; molecular weight 414.3862 Da.	acute alcoholic liver injury	/	Kunming mouse	PI3K/Akt	↓ ALT, AST, TNF-α, IL1-β, IL-6, MDA, p-PI3K/PI3K, p-AKT/Akt, p-(mTOR)/mTOR ↑ SOD, GSH-Px	[99]
others	reduce blood sugar	*C. gigantea*	*C. gigantea* extract	Crude methanol extract.	diabetic	/	Wistar albino rats	/	↓ α-amylase, blood sugar	[9]
		*C. gigantea* *C. Lilacina* *L. fenzlii*	ergosterol	Most abundant characteristic sterol in LC; molecular weight 396.3392 Da.	diabetic kidney injury	/	Kunming mouse	PI3K/Akt/NF-κB	↓ TG, TC, SUA, Scr, p-PI3K, p-Akt, p-NF-κBp65, p-IκB	[25]
	antibechic	*L. fenzlii*	suspension of *L. fenzlii*	Suspension (3 g crude drug/kg).	cough	/	GP	/	extend the incubation period of GP cough	[100]
	antiviral	*L. fenzlii*	*L. fenzlii* extracts	Aqueous and methanolic extracts.	HIV	MT-4	/	/	Antibacterial activity: The IC100 values were 250 and 125 μg/mL respectively, and the CC0 values were 500 and 250 μg/mL respectively.	[101]

Footnote: ↑ means increase; ↓ means decrease. Bax. Bcl-2-associated X protein; Bcl-2. caspase 3. Cysteinyl aspartate specific proteinase-3; B-cell lymphoma 2; WLIP. white linear lipopeptide; Akt. Protein kinase B; CCND1. Cyclin D1; CDK4. Cyclin-dependent kinase 4; PPAR-γ. Peroxisome proliferator-activated receptor gamma; γ-GCS. γ-glutamylcysteine synthetase; ROS. Reactive oxygen species; ERS. Endoplasmic reticulum stress; JNK. c-Jun N-terminal kinase; P-eIF2α. Phosphorylated eukaryotic translation initiation factor 2α; ATF3. Activating transcription factor 3; CHOP. C/EBP homologous protein; GRP78. 78 kDa glucose-regulated protein; HSP90. Heat shock protein 90; c-Myc. Cellular myelocytomatosis oncogene; USP47. Ubiquitin-specific peptidase 47; CGP. C. gigantea polysaccharide; IFN-γ. Interferon gamma; IL-6. Interleukin-6; PI3K. Phosphatidylinositol 3-kinase; mTOR. Mammalian target of rapamycin; PA. Plasminogen activator; PLT. Platelet; GSK3β. Glycogen synthase kinase 3 beta; APTT. Activated partial thromboplastin time; TT. Thrombin time; FIB. Fibrinogen; TNF-α. Tumor necrosis factor-alpha; Arg1. Arginase 1; Mrc1. Mannose receptor C-type 1; TGF-β. Transforming growth factor-beta; Nos2. Nitric oxide synthase 2; VEGFA. Vascular endothelial growth factor A; CGPA1. C. gigantea polysaccharide 1; CPSM. crude polysaccharide from submerged mycelia; γ-GT. γ-glutamyl transferase; SOD. Superoxide dismutase; TG. Triglyceride; TC. Total cholesterol; SUA. Serum uric acid; Scr. Serum creatinine; NF-κB. Nuclear factor-kappa B; HIV. Human Immunodeficiency Virus.

## Data Availability

The original contributions presented in this study are included in the article. Further inquiries can be directed to the corresponding author.

## Abbreviations

The following abbreviations are used in this manuscript:

LC	*Lasiosphaera calvatia*
TCM	Traditional Chinese medicine
Mn	Manganese
Fe	Iron
Mg	Magnesium
Ca	Calcium
K	Potassium
Zn	Zinc
P	Phosphorus
Cr	Chromium
Se	Selenium
CGP	Calvatia gigantea polysaccharide
WLIP	white linear lipopeptide
γ-GCS	γ-glutamylcysteine synthetase
ROS	reactive oxygen species
ER	endoplasmic reticulum
GSH	glutathione
TopoI	topoisomerase I
HSP-90	heat shock protein 90
NK	Natural killer
ERK	extracellular signal-regulated kinase
IFN-γ	Interferon-gamma
PT	prothrombin time
APTT	activated partial thromboplastin time
TT	thrombin time
FIB	fibrinogen
IL-1β	Interleukin-1 beta
TNF-α	Tumor Necrosis Factor-alpha
MIC	minimum inhibitory concentration
VEGF	vascular endothelial growth factor
PEDF	pigment epithelium-derived factor
MDA	malondialdehyde
PA	prealbumin
SOD	superoxide dismutase
TB	total bilirubin
CPSM	crude polysaccharide from submerged mycelia
TG	triglyceride
TC	total cholesterol
SUA	serum uric acid
Scr	serum creatinine

## References

[1] Larsson E., Jeppson M. (2008). Phylogenetic relationships among species and genera of Lycoperdaceae based on ITS and LSU sequence data from north European taxa. Mycol. Res..

[2] Yang X., Duan S., Li M., Li D., Yang R., Zhang S., Xu T., Li W., Zhou H., Zhao C. (2025). A new genus and three new species of Lycoperdaceae (Agaricales) from Southern China revealed by molecular phylogeny and taxonomy. MycoKeys.

[3] de Mattos-Shipley K.M., Ford K.L., Alberti F., Banks A.M., Bailey A.M., Foster G.D. (2016). The good, the bad and the tasty: The many roles of mushrooms. Stud. Mycol..

[4] Cicha-Jeleń M., Muszynska B., Kala K., Sulkowska-Ziaja K. (2024). Medicinal Potential of the Giant Puffball Mushroom *Calvatia gigantea* (Agaricomycetes): A Review. Int. J. Med. Mushrooms.

[5] Pharmacopoeia Commission of the Ministry of Public Health (2025). Chinese Pharmacopoeia.

[6] Zhang X., Zhang Z., Xu Y., Luo J., Shen Z., Liang H., Zeng Y., Liu W., Zheng C., Li J. (2025). Mechanism of Action and Efficacy of Wu-Hua-Yan-Xiao in the Treatment of Pediatric Acute Pharyngitis Based on Network Pharmacology and Experimental Validation. Drug Des. Dev. Ther..

[7] Tu P., Tian R., Lu Y., Zhang Y., Zhu H., Ling L., Li H., Chen D. (2020). Beneficial effect of Indigo Naturalis on acute lung injury induced by influenza A virus. Chin. Med..

[8] Tian R., Zhu H., Lu Y., Shi X., Tu P., Li H., Huang H., Chen D. (2022). Therapeutic Potential of 2-Methylquinazolin-4(3H)-one as an Antiviral Agent against Influenza A Virus-Induced Acute Lung Injury in Mice. Molecules.

[9] Ogbole O.O., Nkumah A.O., Linus A.U., Falade M.O. (2019). Molecular identification, in vivo and in vitro activities of *Calvatia gigantea* (macro-fungus) as an antidiabetic agent. Mycology.

[10] Wang X., Wang G., Tao J., Guo Z., Xu G., Li J., Kang J., Zuo Q., Liu H., Li Q. (2025). Comparative analysis of mitochondrial genomes in lycoperdaceae fungi reveals intron dynamics and phylogenetic relationships. BMC Genom..

[11] Krakhmalnyi M., Isikhuemhen O.S., Jeppson M., Wasser S.P., Nevo E. (2023). Species Diversity of Lycoperdaceae (Agaricales) in Israel, with Some Insights into the Phylogenetic Structure of the Family. J. Fungi.

[12] Yue H.L., Yang Z., Long F.X., Huang W.Q., Yang B., Zhang Z., Tang D.X. (2020). Research on the Medication Regulations of Miao Medicine in Anti-Tumor Treatment. Liaoning J. Tradit. Chin. Med..

[13] Zhang C.H., Man D., Wu G.D., Li Z.H., Zhao D.D., Liu Y., Li W.H. (2015). Protection, exploitation and utilization states of specialized Mongolian folk medicine resources and related development strategy. China J. Chin. Mater. Medica.

[14] Hess W.M., Bushnell J.L., Weber D.J. (1972). Surface structures and unidentified organelles of *Lycoperdon perlatum* Pers. basidiospores. Can. J. Microbiol..

[15] Bi Y.Q., Wang A.X., Bao H.Y., Meng W.W., Zhang C.H., Li Y.H., Zhan L.Z. (2023). Herbal Textual Research on Lasiosphaera Calvatia in Famous Classical Formulas. Chin. J. Exp. Tradit. Med. Formulae.

[16] Xiao X., Kennelly J.P., Ferrari A., Clifford B.L., Whang E., Gao Y., Qian K., Sandhu J., Jarrett K.E., Brearley-Sholto M.C. (2023). Hepatic nonvesicular cholesterol transport is critical for systemic lipid homeostasis. Nat. Metab..

[17] Kivrak I., Kivrak S., Harmandar M. (2016). Bioactive Compounds, Chemical Composition, and Medicinal Value of the Giant Puffball, *Calvatia gigantea* (Higher Basidiomycetes), from Turkey. Int. J. Med. Mushrooms.

[18] Wang X.Q. (2007). Study on Secondary Metabolites and Antitumor Activity of Lasiosphaera fenzlii.

[19] Wang L.W., Wu S. (2017). Research on the Chemical Components of Lasiosphaera fenzlii. J. Qiqihaer Univ. Med. Sci..

[20] Cui L. (2006). Study on the Chemical Composition and Antitumor Activity of Lasiosphaera fenzlii.

[21] Ling Z.N., Jiang Y.F., Ru J.N., Lu J.H., Ding B., Wu J. (2023). Amino acid metabolism in health and disease. Signal Transduct. Target. Ther..

[22] Kıvrak İ., Kıvrak Ş., Harmandar M. (2014). Free amino acid profiling in the giant puffball mushroom (*Calvatia gigantea*) using UPLC-MS/MS. Food Chem..

[23] Su M.Z., Luo Z., Yan M., Zhao Q.C. (2012). Studies on chemical constituents of *Lasiosphaera fenzlii*. Chin. Tradit. Herb. Drugs.

[24] Xiang C.K., Su S.L., Guan S.J., Huang H.P., Wang Q., An F.L., Chen Y.C., Wu H.Y., Zhu S. (2016). Determination of ergosterol in Lasiosphaera Calvatia in various species from different habitats by HPLC. Chin. Tradit. Herb. Drugs.

[25] Li A. (2018). Study on Puffballs Resources in Jilin Province and Material Basis of Pharmacodynamics.

[26] Cui L., Song S.L., Sun L.R. (2006). Study on the Chemical Components of *Lasiosphaera fenzlii* and Preliminary Screening of Its Antitumor Activity. J. Chin. Med. Mater..

[27] Gao Y.J., Zhao Y.J., Min P., Shi G.B., Yan M. (2010). Research on the Chemical Components of *Lasiosphaera fenzlii*. Chin. J. Med. Chem..

[28] Zhang S.Y., Xu L.T., Li A.X., Wang S.M. (2015). Effects of Ergosterol, Isolated from Scleroderma Polyrhizum Pers., on Lipopolysaccharide-Induced Inflammatory Responses in Acute Lung Injury. Inflammation.

[29] Chen H., Chen D.Q., Li Q.F., Li P.F., Chen H., Zhao Y.Y. (2014). Research Progress on Pharmacological Activities, Pharmacokinetics and Content Determination of Ergosterol. China J. Chin. Mater. Medica.

[30] Wang X.Q., Sun L.R. (2007). Research on the Chemical Components of the Traditional Chinese Medicine “*Lasiosphaera fenzlii*”. Nat. Prod. Res. Dev..

[31] Jin X.Q., Wang L.S., Cheng D.Y., Yi X.L., Lv J.S. (1998). Research on the Chemical Components of Calvatia gigantea. Chin. Tradit. Herb. Drugs.

[32] Nobuo K., Setsuko S.M. (1994). Steroids from *Calvatia cyathiformis*. Phytochemistry.

[33] Zeng Q., Singh R., Ye Y., Cheng S., Fan C., Zeng Q. (2022). Calvatia Lilacina Extracts Exert Anti-Breast-Cancer Bioactivity through the Apoptosis Induction Dependent on Mitochondrial Reactive Oxygen Species and Caspase Activation. Nutr. Cancer.

[34] Gao Y.J. (2009). Research on the Hemostatic Active Components and Fingerprint Pattern of the Lasiosphaera fenzlii.

[35] Wu C.L., Meng C.L. (2009). GC-MS Analysis of Monosaccharide Composition in the Polysaccharide from the Puffball Medicinal Fungus. J. Chang. Med. Coll..

[36] Meng J., Fan Y., Su M., Chen C., Ren T., Wang J., Zhao Q. (2014). WLIP derived from *Lasiosphaera fenzlii* Reich exhibits anti-tumor activity and induces cell cycle arrest through PPAR-γ associated pathways. Int. Immunopharmacol..

[37] Meng Y.F., Yang G.L., Zhou X.F. (1990). Studies on Polysaccharide from Puffball. J. Lanzhou Univ..

[38] Huang K., Li Z.X., Deng Y.K., Wu C.L., Nie Y. (2008). Isolation, purification and characterization of polysaccharides from Calvatia gigantea. West China J. Pharm. Sci..

[39] Zhao Y.S., Wang J.P., Song A.R., Wang N.N., Han Z.J., Sun X.B. (2012). Polysaccharide Extraction from Calvatia Gigantea and its in Vitro Anti-tumor Activity. Chin. J. Mod. Appl. Pharm..

[40] Meng J.J., Fan Y.H., Zhao Y.P., Bai J.P., Zhao Q.C. (2013). Determination of White-linear-inducing Principle in *Lasiosphaera fenzlii* Reich By HPLC. Front. Pharm. Sci..

[41] Kim D.S., Jeong H.J., Bhat K.P., Park S.Y., Kang S.H., Yoo E.H., Lee M., Lee H.W., Krueger R.J., Kim D.S. (2000). Aromatase and sulfatase inhibitors from *Lepiota americana*. Planta Medica.

[42] Hou Z. (2014). Study on the Inhibition of Calvacin on SGC-7901 Cell Proliferation.

[43] Ma X., Fan L., Mao F., Zhao Y., Yan Y., Tian H., Xu R., Peng Y., Sui H. (2018). Discrimination of three Ephedra species and their geographical origins based on multi-element fingerprinting by inductively coupled plasma mass spectrometry. Sci. Rep..

[44] Liu Y., Zhao L., He X., Shen Y., Wang N., Hu S., Xu J., Zhao Q., Zhang Q., Qin L. (2023). Jintiange proteins promote osteogenesis and inhibit apoptosis of osteoblasts by enhancing autophagy via PI3K/AKT and ER stress pathways. J. Ethnopharmacol..

[45] Yue L.D., Du L.J., Yang J.N., Zhang Z.L. (2021). Determination and analysis of Inorganic Elements Contents in different varieties of Lasiosphaera seu Calvatiaby ICP-MS and PCA. Asia-Pacific. Tradit. Med..

[46] Zang E.H., Cui H.Y., Bi Y.Q., Liu Y.C., Liu Q., Zhang C.H., Li Y.H., Zhan Z.L. (2024). Research Progress on Chemical Components and Pharmacological Effects of Puffball Species Medicinal Herbs. Chin. Tradit. Pat. Med..

[47] You Y., Bao H.Y. (2011). Antibacterial activities and volatile oil component analysis of extracts of Calvatia gigantea fruiting bodies in different maturity period. Mycosystema.

[48] Xu S.N., Si P., Gao Y.Q., Liu J.H., Yang G.C. (2012). GC-MS Analysis of Volatile Constituents of *Lasiosphaera fenzlii*. Chin. J. Exp. Tradit. Med. Formulae.

[49] Xiang C.K., Guan S.J., Wang Q., An F.L., Chen Y.C., Wu H.Y. (2016). GC-MS analysis for the volatile constituents of *Lasiosphaera calvatia* extracted by different extraction methods. Hebei Med. J..

[50] Li J.Y. (2023). Authenticity Evaluation of Traditional Chinese Medicine Puffballs and Comparative Study on Their Hemostatic Activities.

[51] Hussain S.T., Muhammad S., Khan S., Hussain W., Pieroni A. (2023). Ethnobotany for food security and ecological transition: Wild food plant gathering and consumption among four cultural groups in Kurram District, NW Pakistan. J. Ethnobiol. Ethnomed..

[52] Eroğlu C., Seçme M., Atmaca P., Kaygusuz O., Gezer K., Bağcı G., Dodurga Y. (2016). Extract of Calvatia gigantea inhibits proliferation of A549 human lung cancer cells. Cytotechnology.

[53] De W.L. (2011). Five trillion basidiospores in a fruiting body of Calvatia gigantea. Mycosphere.

[54] Jiang S., Luo G.M., Xu Z.Q., Guan Q.Y. (2024). Quality Status and Analysis of Lasiosphaera Calvatia and its Decoction Pieces. J. Strait Pharm..

[55] Li W.Q., Wen G.Q., Tan G.Y., Zhou M.X., Lin K.X., Qian Z.M. (2025). Advances in quality standards and control of fungal Chinese medicine. Chin. J. Pharm. Anal..

[56] Gao W., Lian Y.F., Yan S.M., Xing J.Y., Wen J.Y., Liu S.J. (2024). Research on the identification of different origin species of Lasiosphaera Calvatia using the systematic identification method. J. Chin. Med. Mater..

[57] Zhang J.L., Huang Y.H., Song M., Ren Y.Y., Zhang M.T., Liu X., Sun W., Chen S.L. (2016). Identification of Lasiosphaera Calvatia and Its Adulterants Using ITS Barcode. World Chin. Med..

[58] Krüger D., Gargas A. (2008). Secondary structure of ITS2 rRNA provides taxonomic characters for systematic studies—A case in Lycoperdaceae (Basidiomycota). Mycol. Res..

[59] Morana O., Wood W., Gregory C.D. (2022). The Apoptosis Paradox in Cancer. Int. J. Mol. Sci..

[60] Wu J.Y., Chen C.H., Chang W.H., Chung K.T., Liu Y.W., Lu F.J., Chen C.H. (2011). Anti-Cancer Effects of Protein Extracts from *Calvatia lilacina*, Pleurotus ostreatus and *Volvariella volvacea*. Evid. Based Complement. Altern. Med..

[61] Tsay J.G., Chung K.T., Yeh C.H., Chen W.L., Chen C.H., Lin M.H., Lu F.J., Chiou J.F., Chen C.H. (2009). *Calvatia lilacina* protein-extract induces apoptosis through glutathione depletion in human colorectal carcinoma cells. J. Agric. Food Chem..

[62] Calcinotto A., Kohli J., Zagato E., Pellegrini L., Demaria M., Alimonti A. (2019). Cellular Senescence: Aging, Cancer, and Injury. Physiol. Rev..

[63] Gao X., Leone G.W., Wang H. (2020). Cyclin D-CDK4/6 functions in cancer. Adv. Cancer Res..

[64] Nilkhet S., Vongthip W., Lertpatipanpong P., Prasansuklab A., Tencomnao T., Chuchawankul S., Baek S.J. (2024). Ergosterol inhibits the proliferation of breast cancer cells by suppressing AKT/GSK-3beta/beta-catenin pathway. Sci. Rep..

[65] Gao J., Wang L.W., Zheng H.C., Damirin A., Ma C.M. (2016). Cytotoxic constituents of *Lasiosphaera fenzlii* on different cell lines and the synergistic effects with paclitaxel. Nat. Prod. Res..

[66] Wu C.L., Wan B., Li J.N. (2016). Isolation, Purification of Acidic Polysaccharide from Calvatia Gigantea and Its Anti-S180 Sarcoma Activity. J. Changzhi Med. Coll..

[67] Mellman I., Chen D.S., Powles T., Turley S.J. (2023). The cancer-immunity cycle: Indication, genotype, and immunotype. Immunity.

[68] Kiel K., Piranlioglu R., Godlewski J., Bronisz A. (2025). Harnessing immunotherapy: Cancer vaccines as novel therapeutic strategies for brain tumor. Front. Immunol..

[69] Onesti J.K., Guttridge D.C. (2014). Inflammation based regulation of cancer cachexia. BioMed Res. Int..

[70] Zhang Z.B., Li H.B. (2019). The inhibitory effect of puffball polysaccharides on tumor growth in breast cancer-bearing mice and its possible mechanism. Mod. Immunol..

[71] Dowling M.B., Smith W., Balogh P., Duggan M.J., MacIntire I.C., Harris E., Mesar T., Raghavan S.R., King D.R. (2015). Hydrophobically-modified chitosan foam: Description and hemostatic efficacy. J. Surg. Res..

[72] He X.R., Fan P.C., Li M.X., Zhang Q.L., Jia Z.P., Zhang R.X. (2010). Advances in Study of Traditional Chinese Hemostyptic Drugs and Related Mechanisms. Chin. J. Exp. Tradit. Med. Formulae.

[73] Yang J.N., Zhang Z.L., Chen J.J., Zhang Y., Li Q.S., Lin X.M., Yan M.Z. (2020). Comparative Study on Hemostasis and Adsorption Force of Different Parts of *Lasiosphaera fenzlii* Reich. China J. Tradit. Chin. Med. Pharm..

[74] Li J.Y., Bi Y.Q., Bao H.Y., Li Y. (2023). Market Investigation and Hemostatic Activity of Chinese Medicinal Puffball Mushrooms. Acta Edulis Fungi.

[75] Yang A.L., Kan C.G., Han X., Yu S.L. (2021). Clinical Effect of Mabo Ointment on Mixed Hemorrhoids. Acta Chin. Med. Pharmacol..

[76] Yang A.L., Han X., Yu B., Kan C.G., Yu S.L. (2023). Effect of Mabo Ointment on NLRP3/Caspase-1 Inflammatory Pathway and Expression of Inflammatory Factors in Rabbits with Escherichia Coli Infectious Wound. Tradit. Chin. Med. J..

[77] Xiang C.K., Guan S.J., Ma J.J. (2016). Study on the comparison of Calvatia gigantea and Bovistella sinensis lloyd on anti-inflammatory and analgesia effects. Tianjin J. Tradit. Chin. Med..

[78] Yang A.L., Kan C.G., Han X., Yu S.L., Sun F.J. (2022). Effect of Mabo Enema on Cytokines of TNF-α and IL-6 in UC Model Mice. Acta Chin. Med. Pharmacol..

[79] Zhang J.M., Zhang D.S., Jing Y.S. (2021). Therapeutic Effect of Lasiosphaera Calvatia Suppository on Ulcerative Colitis in Mice. J. Chin. Med. Mater..

[80] Li Y., Dong T., He T., Zhao Y., Yang C., Xiao H., Mo R., Chen J., Dong J. (2026). Topical application of *Calvatia lilacina* ethanol extract-derived fraction promotes diabetic wound healing. Fitoterapia.

[81] He T., Wang K., Mo R., Guo J., Jiang B., Mu R., Min W., Zhu L., Chen J. (2026). Single-cell RNA sequencing reveals the therapeutic mechanism of *Calvatia lilacina* in promoting wound healing of anal fistula. Chin. Med..

[82] MacLeod A.S. (2019). Bad “Staph” in the Wound Environment of Diabetic Foot Ulcers. Cell Host Microbe.

[83] Zhang F., Wang D.K., Li A.X., Wang S.M. (2014). Study on Anti-inflammatory and Analgesic and Bacteriostasis in Vitro of Calvatia gigantea. Chin. Edible. Fungi.

[84] You Y. (2011). Study on Pharmaceutical of Calvatia gigantea (Batsh ex pers) LIoyd.

[85] Khan S., Fiaz M., Alvi I.A., Ikram M., Yasmin H., Ahmad J., Ullah A., Niaz Z., Hayat S., Ahmad A. (2023). Molecular Profiling, Characterization and Antimicrobial Efficacy of Silver Nanoparticles Synthesized from *Calvatia gigantea* and *Mycena leaiana* against Multidrug-Resistant Pathogens. Molecules.

[86] Paetzold B., Willis J.R., Pereira de Lima J., Knödlseder N., Brüggemann H., Quist S.R., Gabaldón T., Güell M. (2019). Skin microbiome modulation induced by probiotic solutions. Microbiome.

[87] Ding X., Yang C., Li Y., He T., Xu Y., Cheng X., Song J., Xue N., Min W., Feng W. (2024). Reshaped commensal wound microbiome via topical application of Calvatia gigantea extract contributes to faster diabetic wound healing. Burns Trauma.

[88] Bilgen F., Ural A., Kurutas E.B., Bekerecioglu M. (2019). The effect of oxidative stress and Raftlin levels on wound healing. Int. Wound J..

[89] Li Z.M., Ke C.L., Wang Y.B. (2015). Hepatoprotective Effect and Antioxidant Activity of Crude Polysaccharide from Calvatia gigantea Mycelium Grown in Submerged Culture. Acta Edulis Fungi.

[90] Zhu Y., Yuan S.X., Li B. (2010). Scavenging Effect of Water-soluble Polysaccharide in Purple Bald Mabo on Oxygen Free Radical. J. Anhui Agric. Sci..

[91] Pang L., Wang Y., Zheng M., Wang Q., Lin H., Zhang L., Wu L. (2016). Transcriptomic study of high-glucose effects on human skin fibroblast cells. Mol. Med. Rep..

[92] Yang A.L., Han X., Jing W.C., Kan C.G., Yu S.L. (2023). Effect of Mabo Oil Ointment on Wound Healing and Angiogenesis Related Factors VEGF and PEDF in Rabbits with Chronic Skin Ulcer. Chin. Arch. Tradit. Chin. Med..

[93] He T., Sun P., Liu B., Wan S., Fang P., Chen J., Huang G., Min W. (2022). Puffball spores improve wound healing in a diabetic rat model. Front. Endocrinol..

[94] Meng X., Zhao W., Zhao Y., Guo J., Ding X., Gai Y., Lv H., Wang Y., Chen J. (2025). Isolation and structural identification of the homogeneous polysaccharide CGPA1 from Calvatia gigantea with wound healing effects. Carbohydr. Polym..

[95] Shi Y., Ma Y.H., Men L.H., Liu Y., Zhao W., Zhou Y.L., Liu Z.Y. (2012). The effective components of the Chinese herb puffball in promoting the proliferation of rat fibroblasts. Chin. J. Gerontol..

[96] Ma D. (2013). Effects of Traditional Chinese Medicine Puffball on Proliferation and Collagen Synthesis in Rats Skin Fibroblasts.

[97] Zhang Y., Zhao T.Q., Wang H., Wang S.M. (2020). Effects of Ergosterone on Acute Alcoholic Liver Injury and Gut Microbiota Community Composition in Mice. Chin. J. Mod. Appl. Pharm..

[98] Mu S.S. (2020). Study on the Fermentation Technology, Chemical Constituents and Pharmacodynamics of the Calvatia lilacina.

[99] Su H.X., Hou C.Z., Cheng T., Shi W.D. (2024). β-sitosterol relieving realgar-induced liver injury in mice through PI3K/Akt signaling pathways. Chin. J. Integr. Tradit. West. Med. Liver Dis..

[100] Zuo W.Y., Shang M.K., Chuai X.G. (2004). Observation on the Anti-inflammatory and Antitussive Effects of *Lasiosphoera fenzlii* Reich. J. Henan Univ. Med. Sci..

[101] Ma C.M., Nakamura N., Miyashiro H., Hattori M., Komatsu K., Kawahata T., Otake T. (2002). Screening of Chinese and Mongolian herbal drugs for anti-human immunodeficiency virus type 1 (HIV-1) activity. Phytother. Res..

